# Approaches to locum physician recruitment and retention: a systematic review

**DOI:** 10.1186/s12960-024-00906-z

**Published:** 2024-04-16

**Authors:** Nathan Ferreira, Odessa McKenna, Iain R. Lamb, Alanna Campbell, Lily DeMiglio, Eliseo Orrantia

**Affiliations:** 1 Faculty of Medicine, University of Ottawa, Ottawa, ON K1N 6N5 Canada; 2 Division of Clinical Sciences, Northern Ontario School of Medicine (NOSM) University, Marathon, ON P0T 2E0 Canada; 3 Northern Ontario School of Medicine (NOSM) University, Sudbury, ON P3E 2C6 Canada

**Keywords:** Workforce stability, Health human resources, Recruitment strategies, Retention strategies

## Abstract

A robust workforce of locum tenens (LT) physicians is imperative for health service stability. A systematic review was conducted to synthesize current evidence on the strategies used to facilitate the recruitment and retention of LT physicians. English articles up to October 2023 across five databases were sourced. Original studies focusing on recruitment and retention of LT’s were included. An inductive content analysis was performed to identify strategies used to facilitate LT recruitment and retention. A separate grey literature review was conducted from June–July 2023. 12 studies were retained. Over half (58%) of studies were conducted in North America. Main strategies for facilitating LT recruitment and retention included financial incentives (83%), education and career factors (67%), personal facilitators (67%), clinical support and mentorship (33%), and familial considerations (25%). Identified subthemes were desire for flexible contracts (58%), increased income (33%), practice scouting (33%), and transitional employment needs (33%). Most (67%) studies reported deterrents to locum work, with professional isolation (42%) as the primary deterrent-related subtheme. Grey literature suggested national physician licensure could enhance license portability, thereby increasing the mobility of physicians across regions. Organizations employ five main LT recruitment facilitators and operationalize these in a variety of ways. Though these may be incumbent on local resources, the effectiveness of these approaches has not been evaluated. Consequently, future research should assess LT the efficacy of recruitment and retention facilitators. Notably, the majority of identified LT deterrents may be mitigated by modifying contextual factors such as improved onboarding practices.

**Supplementary Information:**

The online version contains supplementary material available at 10.1186/s12960-024-00906-z.

## Introduction

The shortage of a sustainable and robust physician workforce is a significant healthcare issue across most of the world [1]. In regions that face persistent challenges in physician availability, the continuity of the healthcare system heavily relies on locum tenens (LT) physicians, commonly referred to as “locums”. These healthcare providers work in a temporary capacity to fill vacancies or provide coverage for permanent physicians [2, 3]. Their importance was highlighted during the COVID-19 pandemic as the lack of locums resulted in the suspension of hospital services and emergency department closures due to insufficient staffing [4].

Physician recruitment strategies primarily focus on filling permanent positions with minimal emphasis on attracting locum providers [5]. However, strategies aimed at facilitating the recruitment of permanent physicians may not effectively attract locums given fundamental distinctions in their employment preferences and priorities. LTs, for instance, are motivated by factors such as seeking greater autonomy, working part-time, transitioning into partial retirement, and supplementing income [6, 7]. Their attraction to working as a locum may be due to advantages including reduced administrative burdens, lower workplace stress, and flexibility for maintaining a desired work–life balance [6, 7]. Additional advantages include competitive salaries comparable to permanent positions without a long-term commitment, travel and accommodation stipends, subsidized malpractice insurance, and lower overhead expenses [6, 7].

Governments and communities invest substantial financial resources to attract locums in order to sustain healthcare service delivery [6, 8]. As such, existing research has investigated locum recruitment and retention factors [6, 7, 9, 10]. Despite the important role that locums play in sustaining operational healthcare systems, particularly during periods of health human resources strain, there is a lack of consolidated of evidence on the recruitment of LT physicians. Consequently, there is a need for the synthesis of current research on facilitators used in the recruitment and retention of LTs. This will serve to better inform the development of comprehensive, evidence-based recruitment guidelines tailored specifically to LT physicians. Therefore, this study systematically reviewed existing literature to identify and synthesize the approaches used to recruit locum physicians. Ensuing results will provide valuable guidance to policymakers and healthcare organizations, aiding in the development of evidence-based recruitment policies and practices to address the unique needs of locum physicians.

## Methods

This systematic review was performed in accordance with the Preferred Reporting Items for Systematic Reviews and Meta-Analyses (PRISMA) [11]. This research protocol was registered in the PROSPERO database (CRD42022339666).

### Search strategy

Between April 26th and April 27th, 2022 we performed a systematic search of the electronic databases Ovid MEDLINE, Cochrane Database of Systematic Reviews, PsycINFO, CINAHL and Web of Science-Core Collection. Examples of the medical subject headings (MeSH) applied include “Contract Services”, “Career Choice”, “Personnel Staffing and Scheduling”, “Personnel Loyalty” and “Physician Incentive Plans”. This initial search has since been followed by an updated search in October of 2023 prior to submission for publication. Keywords were used to collect non-indexed material and those terms not captured by MeSH, such as “locum”. No limits were applied to the searches. Articles not available in English were excluded. Secondary research (e.g., meta‐analyses, dissertations, systematic reviews, case reports, commentaries, grey literature) were excluded from the scholarly search. Reference lists of included studies were searched for additional articles. Details of the scholarly search strategy appear in Additional file 1: Appendix S1. This search strategy was developed in collaboration with a librarian and peer-reviewed by a second librarian.

Between June 12th and July 16th, 2023 we performed an iterative systematic hand-search of grey literature. This included public search engines (e.g., Google), grey literature repositories (e.g., OpenGrey), health care quality organizations, and data facilities across five countries, Canada, United States of America, United Kingdom, Australia, and India. Examples of the search terms and headings applied include “Locum”, “Contract”, “Temporary”, and “Locum Physician''. Search parameters were restricted to include only articles published in the year 1990 or later. For database searches information beyond the first 150 or 250 search results were not incorporated in the analysis. For full search histories please see Additional file 1: Appendix S3.

### Selection and screening process

A modified version of the PICO (population, intervention, comparison, outcomes) framework was used (Table 1) [12]. We included original qualitative, quantitative or mixed-methods studies focused on recruitment and retention initiatives specific to locums in any country across clinical settings. There was variability in how studies defined locum physicians (Table 2). Articles focusing on recruitment and retention of non-locum physicians and healthcare workers without an MD designation (with the exception of medical students training in a MD programme) were excluded, including articles that combined both populations in which individual data for locums could not be extracted. Articles that exclusively incorporated the recruitment and retention of locums in the interpretive context such that locum recruitment and retention initiatives were not prospectively mentioned in the study framework or methodology were excluded.

**Table 1:** Eligibility criteria involving PICoS (a modified PICO framework)

PICoS	Inclusion criteria	Exclusion criteria
Population	Physician locums, fill-in physicians, temporary physicians or locum, temporary physicians in training (e.g., medical students, residents, or fellows)	Non-physician healthcare workers (e.g., nurses, physician assistants, occupational therapists, respiratory therapists, optometrists, dentists, audiologists) lacking an accredited medical degree Permanent non-locum physicians
Interest	Recruitment and retention of locum physicians at the individual level	Proxy or composite locum recruitment and retention strategies Indirect or inferred locum recruitment or retention strategies Locum recruitment and retention strategies at the managerial, department, organizational or systemic level
Context	Any country Hospital or clinical setting	Non-healthcare settings (e.g., laboratories, pharmaceutical industries, insurance agencies, mines, biotechnology firms, consultant agencies, or medical consultant agencies)
Study type	Qualitative, quantitative, mixed-methods original research in the English language	Secondary research unavailable in English language

**Table 2:** Included studies’ definition of a locum physician

Author (year)	Locum physician definition
Simon & Alonzo (2004, 2008) [7, 13]	*2004* A contingent physician contracted to provide clinical services for a defined period of time without obligation for permanent placement *2008* A locum physician is a temporary physician contracted to provide clinical services for a defined period of time without obligation for permanent placement
Rourke et al. (2003) [3]	NR
Jenson et al. (2008) [15]	Sessional general practitioners are a group pf GPs broadly consisting of both salaried GPs and locums and as such, follow diverse career paths and patterns
DiMeglio et al. (2018) [9]	Physicians hired on a temporary basis to fill vacancies in full-time positions
McKevitt et al. (1999) [16]	A physician who provides care for patients in the absence of permanent physicians
Woloschuk & Tarrant (2002) [17]	NR
Myhre et al. (2010) [2]	A healthcare provider who is serving as a temporary relief or substitute
Jenson et al. (2006) [14]	“Sessional GPs” encapsulated both salaried and locum (freelance) GPs
Rajbangshi et al. (2017) [18]	NR
Theodoulou et al. (2018) [8]	Locum work is multi-faceted and locum physicians cover shifts impromptu or enrol on longer-term contract
Lagoo et al. (2020) [19]	Traveling physicians’ where physicians are sent to practice in hospitals away from their home institution

*GP* general practitioner, *NR* not reported

Retrieved articles were managed using Covidence online systematic review software (Veritas Health Innovation, Melbourne, Australia). Two reviewers independently performed title and abstract screening for relevance. Full texts were then reviewed against eligibility criteria (Table 1). In both stages of screening, discussion was used to resolve disagreements. Remaining discrepancies were resolved by a third reviewer.

### Data extraction and synthesis

Data extraction took place within Covidence using two independent reviewers. A template was developed and piloted for two studies to ensure reviewer agreement prior to utilization. Outstanding conflicts were resolved by a third author. Extraction parameters included study design, participant characteristics, context of locum assignment, and strategies used to recruit and retain locums. Two authors (NF and OM) performed an inductive content analysis to characterize recurring patterns of the locum recruitment and retention strategies discussed in each paper included in the systematic analysis. Following the identification of these strategies, they were grouped into broader, overarching themes relevant to LT recruitment and retention. Methodologic quality of each study was assessed using the Mixed Methods Appraisal Tool (MMAT) [13]; two authors (NF and OM) conducted the appraisal independently and any discrepancies in appraisal were resolved by discussion with a third author (EO). Authors of included studies were contacted if data were missing.

## Results

**Fig. 1:**
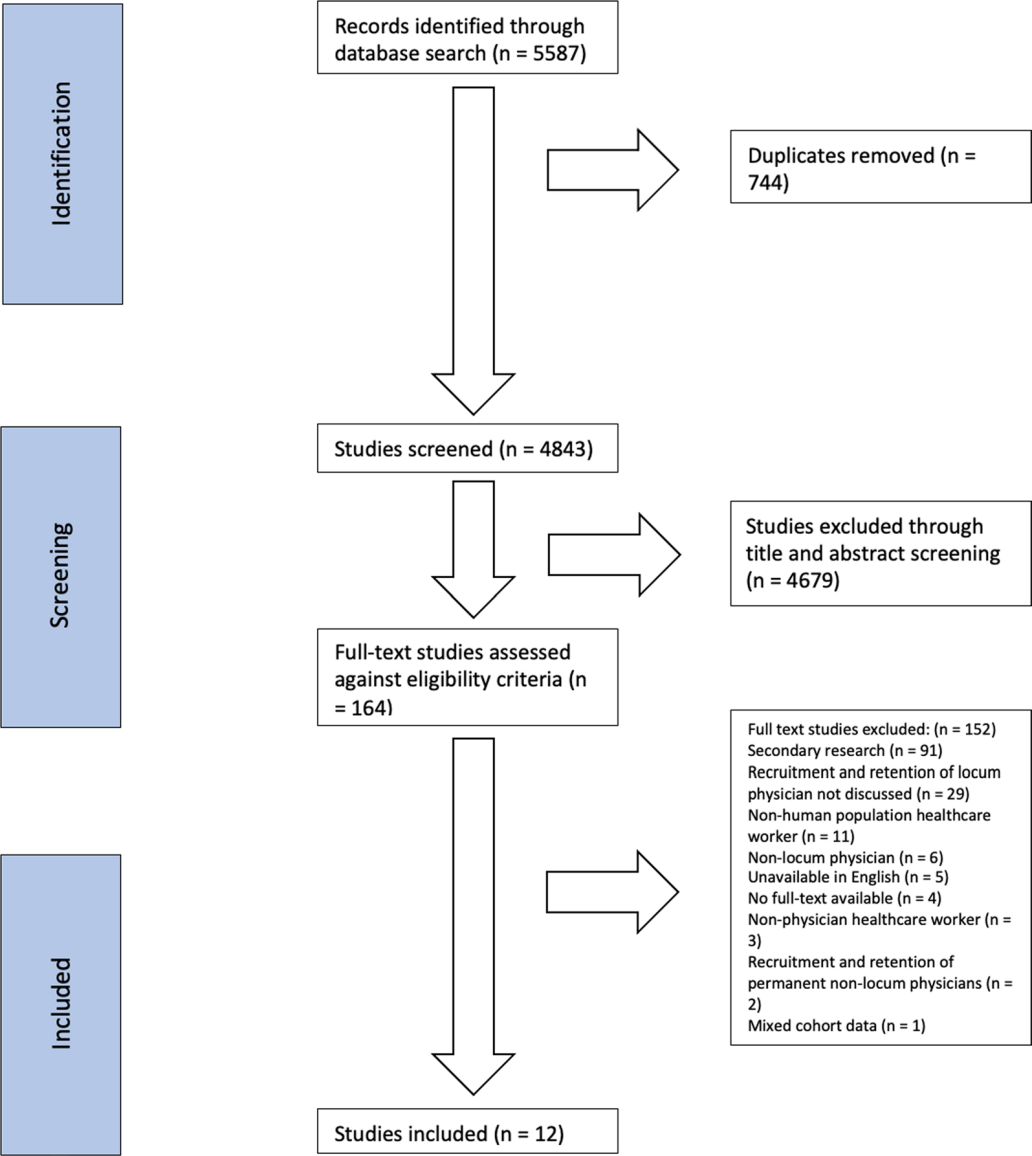
PRISMA flow diagram detailing the selection process

Our initial search identified 5390 citations. After the removal of duplicates (*n* = 812), 4578 studies’ titles and abstracts were screened. Following this stage, 242 articles were screened using full-text, and 230 were excluded from the review. Twelve studies [2, 3, 7–9, 13–19] fulfilled inclusion criteria and were retained for data extraction. The PRISMA flow diagram detailing the screening procedure is displayed in Fig. 1. Articles reporting data from the same participant population at separate time points are reported together.

Study characteristics are summarized in Table 3. Most (*n* = 11, 92%) studies [2, 3, 7–9, 13–15, 17–19] were published within the last 20 years of our search. Four (33%) studies [7, 9, 13, 19] were from the United States and four (33%) [8, 14–16] were from the United Kingdom. A smaller portion (*n* = 3, 25%) [2, 3, 17] originated in Canada. One (8%) study [18] was based in India. Quantitative studies [3, 7, 9, 13, 15–17] (*n* = 7, 58%) were cross-sectional (*n* = 6, 50%) [3, 7, 9, 13, 15, 16] or pre–post study (*n* = 1, 8%) [17] in design. Four (33%) studies [8, 14, 18, 19] used a qualitative design, including semi-structured interviews (*n* = 2, 17%) [18, 19], focus groups (*n* = 1, 8%) [14], and content analysis (*n* = 1, 8%) [8]. One (8%) study [2] adopted a mixed-methods design.

**Table 3:** Study and sample characteristics in the 12 studies included in the review

Author (year)/country	Aim	Design	Setting	Population (specialty breakdown, practice experience)	Sample size (*n*)	Strategies and tools used for locum recruitment	Main findings
Simon & Alonzo (2004, 2008) [7, 13] USA	*2004* To characterize the profile of locum tenens (LT) and the motivation driving the decision to choose this type of practice *2008* To analyse who accepts LT assignments, when in their career they do so, variations in LT practice and what dimensions of satisfaction are important to LTs’ in the context of contingent, nonstandard employment	Cross-sectional	Hospital, clinic, single and multispecialty group practice	Primary care (26.9%), specialist (53.2%), and subspecialist (19.8%) physicians with a mean of 22.6 years practice experience	776	*2004* Respondents had been placed exclusively through LT placement agencies *2008* Placed through LT agencies; arranged for assignments through informal means (unspecified)	*2004* Female LTs were motivated by a flexible work schedule (31%) and were more likely to report locuming as their sole source of income (64%). Male LTs’ primary (62%) motivation was to work part-time and used locum income to augment retirement and pension resources (38%), and as a secondary income (33%) *2008* Locum positions were often sought as an initial position after residency, during a career transition when semi-retired or fully retired. The main reasons for accepting assignments were to: practice part-time (31.5%); obtain a flexible work schedule (21.1%); increase income (15.5%); travel (9.5%); experience new practice settings (8.3%); and avoid administrative responsibilities (2.9%). LT assignments of longer duration were associated with greater problems, including working conditions, transportation, remuneration, and housing
Rourke et al. (2003) [3] Canada	To determine how family medicine residents and practising rural physicians rate potential recruitment and retention solutions for rural practice	Cross-sectional	Broad healthcare settings in rural communities	FM staff physicians (56.8%) and junior and senior FM residents (43.2%)	486^a^	The OMA Locum Program contains a repository of registered physicians to supply locum services in rural areas	Residents rated an alternate payment plan that provides allocated CME time and comprehensive payment plans with guaranteed locum income as the most important recruitment solutions. Rated as “very important” by half or more in at least one of the groups (physicians or residents) included that of practice solutions related to locum provincial licenses, specifically the provision of locum licensure that allows rural physicians to cross provincial borders and provide their services as a locum
Jenson et al. (2008) [15] UK	To calculate the rate of retention of locum GPs and the highest rated recruitment and retention factors	Cross-sectional	PCTs	Primary care physicians (100%) with a mean of 10.4 (SD: 11.5) years practice experience, ranging from 0–49 years	13	NR	Locum recruitment was influenced by work flexibility, availability, environment, and remuneration. Seventy-three (73%) of locum respondents agreed that they would prefer to arrange locum assignments independently. Less than half of locums (46.2%) felt adequately supported with professional development
DiMeglio et al. (2018) [9] USA	To identify clinical duties and remuneration of anesthesiology LT positions using content analysis of recruitment emails	Cross-sectional	Hospitals, surgical and trauma centres	Anesthesiologists (100%)	241^a^	Staffing agencies included the following information about assignments: payment information; licensing requirements; travel reimbursement; case load; and mean daily hours	Most opportunities came from five staffing agencies and information surrounding qualification and licensing was not consistently provided in emails. Majority of emails lacked information for the physician to make an informed decision to accept or decline the assignment. Locum anesthesiologists in the sample had an hourly rate significantly larger than the national average. Some staffing agencies covered licensing expenses (28%), travel (23%), and accommodation costs (20%)
McKevitt et al. (1999) [16] UK	To characterize the motivation behind and experiences of physicians locuming in general practice	Cross-sectional	General practices	Primary care physicians (100%) with a median of two years of practice as a locum	111	Personal network was used to contract physicians	Reasons for pursuing locum assignments included short-term work between posts, experiencing a variety of practices before committing, work–life balance, and becoming part-time after retirement. Drawbacks of LT work included frustration with low status, lack of job security, and difficulty accessing structured training and education
Woloschuk & Tarrant (2002) [17] Canada	To determine the influence of background, gender, and rural educational experience on students' willingness to locum rurally or engage in rural permanent practice	Pre–post study	Rural-based practices	Medical students (100%) with prospective interest in FM training	254^b^	Rural educational experience for students increases likelihood of pursuing a rural locum assignment	Rural educational experiences in clerkship increased the chances of medical students choosing to take on rural locums and cemented novel rural connections
Myhre et al. (2010) [2] Canada	To understand the demographics of family medicine residency graduates choosing locum work, their reasons behind choosing and leaving locum work, and its shortcomings and rewarding aspects	Mixed-methods	Hospitals, general practice, family medicine residency training programmes	Family medicine residency graduates (100%)	155^a^	NR	Most (46.7%) cited practice exploration to increase experience and competence as the main reason to locum. The main reason for leaving a locum post was starting permanent practice (52.1%). Other reasons included desires for stability and patient continuity, and the impacts of personal life changes, financial considerations, and an end of need for exploration. Downsides to locum practice included negotiating contracts, low patient counts, lack of continuity, and working with difficult staff. Rewards of locum practice included flexibility and freedom in practice, increasing experience, and awards associated with seeing patients (unspecified)
Jenson et al (2006) [14] UK	To gain an understanding of the role of a primary care trust (PCT) educational support group in the recruitment and retention of locum physicians	Focus group	Urban PCTs	GPs with a range of 2–40 years practice experience	9^a^	Locum bank arrangements providing administrative support	GP (general practitioner) and locum retention factors included a supportive network of colleagues, assistance keeping up to date, and financial factors. Access to an educational support group was the most impactful factor influencing retention, especially for locums
Rajbangshi et al. (2017) [18] India	To gain insight to the motivation and challenges of rural recruitment and retention of doctors and nurses in India’s states of Meghalaya and Nagaland	Semi-structured interviews	Urban and rural-based district and maternity hospitals, primary and community health centres, and general government service	Generalist (90%) and specialist physicians (10%)	10	NR	Rural background and community ties were strongly associated with decisions to join rural facilities regardless of the type of contract worker (locum physician or nurse). Poor working and living conditions, poor job security, lack of professional development and recognition, low salary, and lack of incentives deterred contractual locum physicians from rural service. Lack of pay parity and job security were unique challenges faced by contractual locum physicians
Theodoulou et al. (2018) [8] UK	To explore the locum doctor landscape, and secondarily to evaluate the implementation of a novel software platform for temporary staff recruitment in the healthcare industry	Content analysis	Workforce planning in British healthcare system	Industry experts (15%), regional based strategy consultants in digital healthcare (15%), executive trust directors (8%), specialty managers (15%) and physicians (46%)	13^c^	Key information (e.g., hourly rates, hospital maps) about locum assignments were shared between hospitals and prospective locums via point of contact. Use of internal staff bank and external agencies. Use of novel software termed “ABG”	Locum physicians were imperative to covering physician shortages, but current recruitment practices were inefficient, lacking transparency and consistency. The financial remuneration of the locum assignment has been said to be a motivator for “pay-centric” physicians. Locum physicians highlighted that this line of work does not allow for career progression and is dissatisfying because of lack of patient continuity of care. Lack of information made available about the LT assignment creates some professional isolation and logistical issues that prevent smooth completion of shifts. Mobile first solutions such as the secure third-party communication (ABG) software was more convenient, transparent, and cost and time effective. Findings enabled the development of an Information Exchange System to compare recruitment methods in healthcare
Lagoo et al. (2020) [19] USA	To (1) better understand the potential areas of risk when a physician is first working in an unfamiliar setting; and (2) develop a tool for surgeon onboarding that can also be further refined to meet the needs of different specialties for the purposes of physicians who are recruited to work at hospitals outside of their home institution, through professional service agreements (PSA)	Semi-structured interviews	Academic medical centres and community hospitals	Family medicine (5%), emergency medicine (5%), OB/GYN (20%), general surgery (45%), anesthesiology (5%), and internal medicine (15%) physicians with most (50%) having greater than 20 years of practice experience and few (15%) with less than 10 years of practice experience	20^a^	NR	Key findings from the aspect of travelling physicians detailed that logistics of providing care in a new setting are not usually integrated into onboarding practices which can limit the ability of the physician to provide efficient and effective care. Lack of interpersonal connections impacts the physicians' ability to acquire help with working in new settings

*CME* continuing medical education, *FM* family medicine, *GP* general practitioner, *LT* locum tenens, *OB/GYN* obstetrics & gynaecology, *OMA* Ontario Medical Association, *NR* not reported, PCT, Primary care trust *PSA* professional service agreements, *SD* standard deviation, *SGP* sessional general practitioner

^a^Indicates that reported data are not specific to locum population and contains a mixed cohort of locum and non-locum physicians

^b^Data refer to non-physician population

^c^Data refer to mixed non-physician and locum physician population

The majority of studies [2, 7–9, 13, 15, 16, 19] (*n* = 8, 67%) specified clinical setting, but did not indicate whether it was rural or urban (Table 3). A variety of specialities were reported among locum populations. The majority (*n* = 9, 75%) [2, 3, 7, 13–16, 18, 19] included primary care physicians, and over half (*n* = 5, 42%) [7, 9, 13, 18, 19] included specialists. Three (25%) studies [7, 13, 19] reported a subspecialist population. Two (17%) studies [2, 17] included physicians in training, with one (8%) [2] involving resident physicians and another (8%) [17] medical students. One (8%) study [8] did not indicate the specialty of the physician population. A total of six (50%) studies [7, 13–16, 19] reported years of physician practice experience.

A diversity of locum recruitment approaches were reported across studies, with some (*n* = 2, 17%) [7, 8] using more than one method. Four (33%) studies [7–9, 13] used a third-party recruitment agency, two (17%) [8, 14] used a locum bank, word-of-mouth, or personal networks [16], informal means [7], and an unspecified novel recruitment software were each reported once [8] (8%). Four studies [2, 15, 18, 19] (33%) did not report a specific method.

### Quality assessment

The 2018 version of the MMAT was used to appraise the quality of retained articles [20]. Overall, nine (75%) of the articles [7, 8, 13, 14, 16–19] met 75–100% of the evaluated criteria, representing high quality. Three (25%) studies [2, 9, 15] met 50–75% of the evaluated criteria, representing moderate quality. Further details regarding the assessment of quality of retained articles appear in Additional file 1: Appendix S2. Grey literature was assessed using the AACODS Checklist [21]. Additional information on the appraisal of grey literature can be found in Additional file 1: Appendix S3 and in the supplemental content titled “Grey Literature Search Strategy, Data Extraction, and Evaluation”.

### Facilitators of locum recruitment and retention

Six locum recruitment and retention themes were identified across retained studies (Table 4). Five overarching themes emerged for factors that facilitated LT recruitment and retention: financial incentives, familial considerations, educational or career-based factors, personal facilitators, and mentorship/clinical support. One theme focused on deterrents of locum work.

**Table 4:** Themes of deterrents and incentives of locum recruitment and retention reported by retained studies

Author (year)	Financial incentives	Familial incentives	Educational/career-based incentives	Personal incentives	Mentorship and clinical back-up support	Deterrents to locum work
Simon & Alonzo (2004, 2008) [7, 13]	*2004* Supplement retirement income for older physicians and increase in income *2008* Increased remuneration, and reimbursement for travel and lodging, cover fees for state medical licensure, and supply malpractice insurance	*2004* NR *2008* Housing (unspecified) and family (unspecified)	*2004* Pre-permanent practice scouting. Career transition and easing into retirement when considered to be semi-retired or retired. Free from administrative responsibilities. Desire to practice on a part-time basis *2008* Pre-permanent practice scouting Facilitate hospital credentialling and verify medical licensure. Career transition and easing into retirement when considered to be semi-retired or retired. Free from administrative responsibilities Desire to practice on a part-time basis	*2004* Locum tourism, to travel and experience a different practice setting. Flexibility of work schedule *2008* Locum tourism, flexible work schedule, facility amenities (unspecified), work conditions (unspecified), overall quality of the facility (unspecified)	NR	NR
Rourke et al. (2003) [3]	OMA Rural Locum Program funds accommodation, travel and guaranteed income for locum physicians (unspecified). Payment assistance for CME	NR	Access to CME and provision of a cross-provincial locum physician medical license	NR	NR	NR
Jenson et al. (2008) [15]	General remuneration for the purposes of recruitment (unspecified)	NR	NR	Flexibility of work schedule, work–life balance, and personal safety (unspecified)	Having known colleagues to work alongside	NR
DiMeglio et al. (2018) [9]	Complimentary assistance with licensing costs, paid travel and lodging, and provision of malpractice coverage	NR	NR	NR	NR	Stress due to uncertainty regarding the requirements of the assignment shown to as a significant drawback for locum tenens physician
McKevitt et al. (1999) [16]	Financial benefits (unspecified)	NR	Avoidance of commitment to a single practice. Allows for transition to retirement or temporary employment between permanent positions Practice task load and low administrative burden including paperwork, being on-call, and business management	Flexibility of work schedule, work–life balance, and locum tourism (ability to visit new places as a locum and travel)	Having familiarity with the practice	Poor job security, work unpredictability and exclusion from the National Health Service pension. Lack of career training opportunities. Perception of inferior professional status of locum by colleagues. Not enough patient continuity of care, as well as frequent travelling
Woloschuk & Tarrant (2002) [17]	NR	NR	Pre-permanent practice scouting allowing the opportunity to assess suitability of the practice and community	Locum tourism	NR	Rural locum work can be demanding for new graduates in their early years of training
Myhre et al. (2010) [2]	Financial benefits (unspecified) and the opportunity to increase income	Flexibility in accommodating family life	Increase clinical competence and skills. Pre-permanent practice scouting. Gain exposure to and develop understanding of running a professional practice. Flexibility in practice change and delaying commitment potential practice	Flexibility to accommodate lifestyle, work–life balance, and flexible work schedule. Time off to travel and participate in locum tourism	NR	Exhaustion. In addition, reasons for departure included the need for practice stability, continuity of care, and lack of patient volume, as well as being accepted by staff at the assignment were also downfalls
Jenson et al. (2006) [14]	Financial factors for improvement of retention (not further specified)	Proximity to family and friends and schooling availability	Career advancement opportunities such as peer-facilitated educational support and help with education for keeping up to date	Post with flexible contracts, structured support (unspecified) and stress relief (unspecified)	Having access to a network of supportive colleagues	Isolation due to inadequate supportive network of colleagues and absence of sense of belonging. Difficulty remaining current as a physician and not feeling part of the ‘mainstream’ education system, including distance from postgraduate centres, and limited accessibility of different types of group education
Rajbangshi et al. (2017) [18]	Increased remuneration for working in difficult conditions known as the “hardship allowance”	NR	NR	NR	NR	Low salary, and lack of equitable pay. Lack of career advancement, professional isolation, poor job security, and working conditions. Inadequate living conditions, difficulty in accessing leave of absences. Displeasure of administrative burdens
Theodoulou et al. (2018) [8]	Hourly rates of locums higher than full-time contracted physicians and pay-centric doctors appear content with such enticing pay rates	NR	Originating from the reality that the early training programmes limit physicians’ exposure to certain specialities, locuming offers the opportunity to attain additional skills	Flexibility of work schedule and work–life balance, specifically logistical flexibility, compatibility with posts, and convenience of the locum assignment	NR	Lack of effective information sharing between physicians and managers to make informed decisions. Instability and desire to attain a secure position. Lack of care continuity. Professional isolation because of the need to adapt to new settings, no immediate “camaraderie” benefits, and native staff resentment about high locum pay. Uncertainty of where to go and who to report to, accessing buildings. Inadequate hospital onboarding practices reported as the most important deterrent to undertaking locum assignments
Lagoo et al. (2020) [19]	NR	NR	NR	NR	Back-up or buddy physician available during the assignment to aid in understanding institutional workflow and culture. Intentional relationship building whereby seasoned colleague meets with the new LT physician several times to ensure comfort in the practice. Possibility of locum physician visiting the new hospital prior to assignment increases familiarity	Poor onboarding practices cause a lack of interpersonal relationship development. Inadequate time set aside to introduce key team members and foster relationships, leading to difficulties asking for help on shift. Information such as EMR basics, email access, and key team members not always provided. Lack of information on how home institution differed from locum assignment institution reduced welcomeness to the new team. Physicians described uncertainty and high stress in providing care in an emergency while in a new setting

*CME* continuing medical education, *NR* not reported, *OMA* Ontario Medical Association

Ten (83%) studies [2, 3, 7–9, 13–16, 18] reported financial incentives with nine individual subthemes identified (Table 5). A significant portion (*n* = 4, 33%) of studies’ [2, 14–16] did not provide specific details about the nature of the financial incentives provided. Four (33%) of the studies’ [2, 7, 8, 13] financial incentives referred to an increase in income. Reimbursement for locum travel and lodging was reported three times (25%) [3, 7, 9]. Reimbursement for medical licensure (*n* = 2, 17%) and provision of malpractice insurance (*n* = 2, 17%) were also reported [7, 9]. Augmented pay for challenging work conditions [18], payment assistance for continuing medical education (CME) [18], supplementation of retirement income [13], and guaranteed income [3] were all reported once (8%) each.

**Table 5:** Subthemes identified under each recruitment and retention incentive theme across the 12 included studies

Financial (*n* = 9)	Familial (*n* = 3)	Educational/career-based (*n* = 13)	Personal (*n* = 5)	Mentorship and clinical back-up support (*n* = 4)	Deterrents (*n* = 19)
Financial factors (unspecified): 4 (33%) Increase income: 4 (33%) Reimbursement travel and lodging: 3 (25%) Reimbursement for state medical licensure: 2 (17%) Provision of malpractice insurance: 2 (17%) Supplement retirement income: 1 (8%) Payment assistance for CME: 1 (8%) Increased remuneration for difficult conditions: 1 (8%) Guaranteed income: 1 (8%)	Accommodate family: 2 (17%) Unspecified familial incentives: 1 (8%) (housing and family) School availability: 1 (8%)	Pre-permanent practice scouting: 4 (33%) Temporary/transitional employment: 4 (33%) Free from administrative responsibilities: 3 (25%) Ease into retirement: 3 (25%) Increase skills and clinical competence: 2 (17%) Avoid commitment: 2 (17%) Desire to take on part-time employment: 2 (17%) Peer-facilitated educational support: 1 (8%) Help with keeping up-to-date: 1 (8%) Access to CME: 1 (8%) Gain exposure to understand running a medical practice: 1 (8%) Facilitate hospital credentialling and medical licensure: 1 (8%) Cross-provincial locum medical license: 1 (8%)	Flexible contracts (suitable availability/work schedule/work–life balance): 7 (58%) Locum tourism: 5 (42%) Unspecified personal incentives reported: 3 (25%) (Stress relief, structured support, amenities, work conditions, overall quality of facility, and personal safety) Compatibility with posts: 1 (8%) Convenience of the assignment: 1(8%)	Network of supportive colleagues: 2 (17%) Being familiar or having a chance to become familiar with the practice (e.g., locum arrives before shift (potentially a day before) to orient themselves)): 2 (17%) Intentional relationship building whereby the seasoned colleague meets with the new LT physician to ensure comfortability in the practice: 1 (8%) Back-up physician available: 1 (8%)	Professional isolation: 5 (42%) Work unpredictability: 4 (33%) Not enough patient continuity of care: 3 (25%) Inadequate onboarding and orientation practices: 2 (17%) Lack of information to make an informed decision about accepting the post: 2 (17%) Demanding locum work: 2 (17%) Poor job security: 2 (17%) Lack of career advancement/training: 2 (17%) Too frequent travelling: 1 (8%) Low patient volume: 1 (8%) Displeasure of administration burdens:1 (8%) Difficulty accessing leave of abscences:1 (8%) Difficult living conditions: 1 (8%) Difficult working conditions:1 (8%) Not included in pension plans: 1 (8%) Lack of equitable pay: 1 (8%) Low salary: 1 (8%) Feeling “distant” from the mainstream education system and difficulty keeping up to date: 1 (8%) Perceptions of inferior professional status by colleagues: 1 (8%)

*CME* continuing medical education

Three (25%) studies [2, 7, 14] reported familial considerations as facilitators to recruitment and retention which included accommodating family (17%) [2, 14], school accessibility (8%) [14], and unspecified (8%) [7]. Furthermore, eight (67%) studies [2, 3, 7, 8, 13, 14, 16, 17] reported education or career-based incentives. A total of 13 subthemes related to educational and career-based factors facilitating recruitment and retention were reported (Table 5), which included pre-permanent practice scouting [2, 7, 13, 17] and temporary or transition in employment [2, 7, 13, 16] both reported four (33%) times. Freedom from administrative responsibilities and transitioning into retirement were reported three (25%) times [7, 13, 16]. Avoiding commitment [2, 16], increasing skills and competencies [2, 8], and a desire to take on part-time employment [7, 13] was reported twice (17%). The remaining career-based facilitators to recruitment and retention were each reported once (8%), including acquiring cross-provincial locum medical licensure [3], facilitation of hospital credentialing and medical licensure [7], gaining exposure to running a medical practice [2], accessing novel CME opportunities [3], assistance with maintaining medical knowledge [14], and accessing peer-facilitated educational support [14].

A total of eight (67%) studies [2, 7, 8, 13–17] reported using personal factors as facilitators of LT recruitment and retention. Within this category, five subthemes were identified (see Table 5). Seven (58%) reported using flexible contracts (e.g., suitable availability, work schedule flexibility, and work–life balance) [2, 7, 8, 13–16]. Having the ability to travel and experience new communities (locum tourism) was reported five (42%) times [2, 7, 13, 16, 17]. Three (25%) studies [7, 14, 15] reported unspecified personal incentives including stress relief [14], structured support [14], facility amenities [7], working conditions [7], personal safety [15], and overall facility quality [7]. Compatibility with post and convenience of the assignment were reported once (8%) each [8].

Four (33%) studies [14–16, 19] reported recruitment and retention facilitators involving mentorship and clinical support with four subthemes. Having a network of supportive colleagues [14, 15] and a chance to become familiar with the practice before arrival [16, 19] were reported twice (17%) each. Intentional relationship building, whereby the seasoned colleague met with the incoming locum to ensure comfort in the practice was reported once (8%) [19]. Availability of a back-up physician for support was reported once (8%) [19].

A total of eight (67%) studies [2, 8, 9, 14, 16–19] addressed deterrents of or barriers to locum work, encompassing a total of 19 reported subthemes. Professional isolation (*n* = 5, 42%) [2, 8, 14, 18, 19] was reported most frequently followed by work unpredictability (33%) [2, 8, 16, 19]. Insufficient patient continuity of care was reported three (25%) times [2, 8, 16]. The following deterrents/barriers were each reported twice (17%): inadequate employee onboarding and orientation [8, 19], demanding locum work [2, 17], poor job security [16, 18], lack of information to make an informed decision about accepting the job post [8, 9], and a lack of career advancement [16, 18]. The following deterrents/barriers were reported just once (8%): excessive travelling [16], low patient volume [2], administrative burden [18], difficulty accessing time-off [18], inadequate housing [18], challenging working conditions [18], exclusion from pension plans [16], lack of equitable pay [18], low salary [18], feeling distanced from CME and limitations in staying up-to-date [14], and perceptions of inferior professional status by colleagues [16].

### Facilitators of locum recruitment and retention within grey literature

Grey literature findings closely mirrored the facilitators and deterrents found in peer-reviewed literature. A notable exception captured in the ‘education and career’ theme involved the potential benefits of implementing a national physician licensure, which was absent in the primary literature but present in nearly a third (*n* = 27, 26%) of the grey literature.

## Interpretation

We identified 12 English language studies that explored the recruitment and retention of locums in Canada, USA, UK, and India over a 30-year period. Finance, education, and personal factors were the most used LT recruitment strategies while family considerations and clinical/mentorship support were less frequently cited. However, almost all studies [2, 3, 7, 8, 13–16] (*n* = 8, 67%) reviewed reported using a combination of these recruitment approaches. While there is a paucity of evidence on whether employing multiple approaches leads to improved LT recruitment, utilizing a range of methods may still be a reasonable strategy. This approach prevents organizations from becoming overly reliant on a single approach and enables them to adapt their strategy more easily as required to maintain LT recruitment, retention and service. Further, as physicians choose locum positions based on different priorities, utilizing multiple strategies provides a range of incentives with wider appeal.

Across the five LT recruitment strategies, the diverse range of unique approaches used indicates there is no one-size-fits-all method. This suggests that organizations develop their own specific approach tailored to their available resources, location, and the anticipated needs of the LT physicians they aim to recruit. For instance, certain recruitment strategies incentives such as back-up availability, network of supportive colleagues and access to CME may not be feasible for some organizations given their size, location, and resource constraints. This may lead to the development of alternative recruitment approaches and/or increased emphasis on other strategies. Notably, we found that common recruitment and retention strategies used elsewhere, such as providing competitive salaries, were extensively used in the recruitment and retention of locums. However, approaches that seem to be specifically designed to address the unique requirements and preferences of locum physicians were also employed, such as offering reimbursements for travel and accommodation, providing support for family-related needs, offering flexible scheduling, and facilitating access to leisure activities. Although the effectiveness of these strategies is poorly defined, their implementation suggests that organizations recognize that conventional recruitment and retention approaches, effective in the broader health workforce, may not adequately address the unique aspects and challenges associated with the transient and temporary nature of locum work. For example, incentives like competitive compensation, while valued, might not be as appealing to those seeking the flexibility of short-term work assignments or lifestyle benefits. Therefore, acknowledging the appeal of locum work, creating incentives that emphasize these benefits, and addressing the related challenges are likely to enhance recruitment and retention efforts.

The finding that showed sites employed a wide range of recruitment and retention approaches highlights the complexity of this process. However, implementing such a wide range of strategies makes it challenging to identify the most effectives. Consequently, future work should identify optimal recruitment strategies within diverse health contexts and organizational structures. This would enable organizations to streamline their approach, maximizing recruitment success while efficiently utilizing their resources. This may be particularly valuable in resource poor healthcare environments where strategic asset allocation is essential.

Numerous factors were cited as deterrents of locum work, indicating that physicians’ decision to work as a LT is influenced by a variety of considerations. Although some of the cited deterrents were addressed by recruitment strategies, it is unclear whether these approaches were effective. In the studies reviewed, professional isolation and work assignment predictability were the two most cited deterrents to locum work appearing in 42% and 33% of studies, respectively. As temporary workers, there are inherent challenges in developing rapport with colleagues. Moreover, providing coverage introduces uncertainties regarding work schedules and conditions (e.g., hours worked, frequency and duration of assignments). Together these factors can contribute to lower job satisfaction, which may result in a decreased willingness to work as a locum. As a result, recruitment strategies should consider measures to address these deterrents. The wide range of deterrents emphasizes the importance for healthcare organizations to adopt comprehensive recruitment strategies that recognize and respond to the various unique needs of LT physicians. Further, many of these deterrents may be addressed by improving locum onboarding and job conditions, such as enhancing infrastructure quality and minimizing social isolation.

It is important to recognize information on locum recruitment and retention extends beyond peer-reviewed articles to include the grey literature. These non-academic resources contain potential insights into practical approaches for recruitment and retention, thus underscoring the need to evaluate the grey literature in this field. Interestingly, our review of the grey literature generally aligns with the facilitators and deterrents of locum work identified in this systematic review apart from support for a national physician license. Such a measure would enhance the portability of licensure, allowing improved mobility of physicians across regions, reducing administrative burdens and the time required for obtaining proper licensing, hospital privileges, and contractual agreements. This, in turn, may reduce barriers to locum recruitment and more effectively facilitate the transition of locums to their temporary place of practice. This finding, which was not identified in the systematic review, again reiterates the importance of assessing the grey literature to gain a comprehensive understanding on the current strategies being used for recruiting and retaining locum physicians.

Importantly, the success of LT physician recruitment relies on a collaborative effort that extends beyond responsibility of individual healthcare organizations. This is particularly important considering that facilitators of LT recruitment and retainment, such as remuneration, fall beyond the scope of health teams. Therefore, the various stakeholders in health human resources, including educational institutions, regulatory bodies, and professional associations, all play a role in LT recruitment efforts. Recognizing and embracing this shared responsibility will be crucial in fostering a robust and sustainable healthcare workforce that incorporates LT physicians.

## Limitations

In the systematic and grey literature reviews, a comprehensive set of keywords related to locum recruitment and retention were used (as detailed in Additional file 1: Appendices S1 and S3). However, some search terms, such as region-specific terminology used to describe locums, were not included. As a result, it is possible that relevant resources may have been missed during the literature search. However, the use of diverse keywords related to locum recruitment and retention would have captured relevant studies thus reducing the likelihood that relevant resources were missed. As described in the literature search strategies, date limitations were applied to both the systematic and grey literature searches, and only select databases were searched. Therefore, there is a possibility that relevant publications or grey literature produced outside of these date ranges or databases might have been missed. The quality of synthesized evidence was moderate as most of the retained quantitative studies were cross-sectional [3, 7, 9, 13, 15, 16] (50%) or mixed-methods [2] (8%). Only one study adopted a pre–post study design [17], which is fraught with internal validity issues. Remaining studies [8, 14, 18, 19] were qualitative and not inherently generalizable to broad populations. None of the studies were intervention-based, making it difficult to draw conclusions about the effectiveness of various recruitment and retention strategies. Inconsistent reporting on locum (LT) gender limited conclusions regarding differences in motivations for LT practice. Geographies of included studies reported were likely influenced by the methodological choice to include English only articles, limiting the generalizability of the presented findings to other regions. Further, the mix of qualitative and quantitative sources make it challenging to comprehend the cumulative size of the physician population raising each issue, and the relative significance of each issue compared to others.

## Conclusions

This systematic review synthesized existing knowledge pertaining to international locum physician recruitment and retention strategies. Locum physicians are essential to the delivery of quality healthcare services across Canada and other parts of the world. We demonstrate that organizations employ five main LT recruitment strategies and deploy these in a variety of ways. Though these may be incumbent on local resources, more concerning is that the effectiveness of these approaches has not been tested. Given the present financial challenges within the global healthcare landscape there is a need to better understand recruitment and retention strategies of LTs so this limited resource can be used most effectively. Findings merit future research into the effectiveness of LT recruitment approaches via prospective methodologies.

### Supplementary Information

**Additional file 1. Appendix S1**: Search Strategy. **Appendix S2**: Mixed-methods Appraisal Tool (MMAT) quality assessment of included studies. **Appendix S3**: Grey Literature Search Strategy, Data Extraction, and Evaluation.

## Data Availability

All data generated or analysed during this study are included in this published article [and its supplementary information files].
