# Supplementary material for: Approaches to locum physician recruitment and retention: a systematic review
Source: Hum Resour Health. 2024 Apr 16;22:24. doi: 10.1186/s12960-024-00906-z (PMC11020646; doi:10.1186/s12960-024-00906-z)
Supplement: Supplementary file 1 — Additional file 1. Appendix S1: Search Strategy. Appendix S2: Mixed-methods Appraisal Tool (MMAT) quality assessment of included studies. Appendix S3: Grey Literature Search Strategy, Data Extraction, and Evaluation. [file 12960_2024_906_MOESM1_ESM.docx]

**Appendix S1 - Search Strategy**

Between April 26th and April 27th, 2022 we performed a systematic search of the electronic databases Ovid MEDLINE (1946-Present, including ahead of print, in-process, in-data-review and other non-indexed citations), Cochrane Database of Systematic Reviews, PsycINFO, CINAHL and Web of Science - Core Collection (1975-Present). This initial search has since been followed by an updated search in October of 2023 prior to submission for publication. Examples of the medical subject headings (MeSH) applied include “Contract Services”, “Career Choice”, “Personnel Staffing and Scheduling”, “Personnel Loyalty” and “Physician Incentive Plans”. Keywords were used to collect non-indexed material and those terms not captured by MeSH, such as “locum”. No limits were applied to the searches. For full search histories please see Supplemental Content. This search strategy was developed in collaboration with the librarian on the team who completed each database search. A second librarian peer-reviewed the search (see acknowledgements).

**Supplemental Content**

**Ovid MEDLINE(R) and Epub Ahead of Print, In-Process, In-Data-Review & Other Non-Indexed Citations, Daily and Versions(R)**

Database: Ovid MEDLINE(R) and Epub Ahead of Print, In-Process, In-Data-Review & Other Non-Indexed Citations, Daily and Versions

1 locum*.mp.

2 Contract Services/

3 (contract adj2 service*).mp.

4 Career Choice/

5 "Personnel Staffing and Scheduling"/

6 exp Physicians/sd [Supply & Distribution]

7 exp Physicians/px [Psychology]

8 intention/

9 Motivation/

10 exp Workforce/

11 Job Satisfaction/

12 "Attitude of Health Personnel"/

13 Employment/

14 Personnel Turnover/

15 Personnel Management/

16 personnel loyalty/

17 Employee Incentive Plans/

18 Physician Incentive Plans/

19 Personnel Selection/

20 (staff* adj2 turnover*).mp.

21 ((job or work) adj2 satisfaction).mp.

22 ((work adj2 place*) or workplace*).mp.

23 (employment or employing).mp.

24 (hire or hiring).mp.

25 (personnel adj2 loyalty).mp.

26 (choice* or motivat* or recruit* or retention or retain* or intention* or attract* or migrat* or mobility or workforce* or disincentive* or incentive* or expectation* or staffing).mp.

27 ((labour or labor) adj2 suppl*).mp.

28 ((labour or labor) adj2 force*).mp.

29 1 or 2 or 3

30 4 or 5 or 6 or 7 or 8 or 9 or 10 or 11 or 12 or 13 or 14 or 15 or 16 or 17 or 18 or 19 or 20 or 21 or 22 or 23 or 24 or 25 or 26 or 27 or 28

31 29 and 30

**PsycINFO**

1 locum*.mp.

2 (contract adj2 service*).mp.

3 exp occupational choice/

4 work scheduling/

5 physicians/

6 exp Physicians/

7 motivation/

8 job satisfaction/

9 exp health personnel attitudes/

10 employee turnover/

11 loyalty/

12 exp incentives/

13 personnel selection/

14 (staff* adj2 turnover*).mp.

15 ((job or work) adj2 satisfaction).mp.

16 (work adj2 place*).mp.

17 (employment or employing).mp.

18 (hire or hiring).mp.

19 (personnel adj2 loyalty).mp.

20 (choice* or motivat* or recruit* or retention or retain* or intention* or attract* or migrat* or mobility or workforce* or disincentive* or incentive* or expectation* or staffing).mp.

21 ((labour or labor) adj2 suppl*).mp.

22 ((labour or labor) adj2 force*).mp.

23 1 or 2

24 3 or 4 or 5 or 6 or 7 or 8 or 9 or 10 or 11 or 12 or 13 or 14 or 15 or 16 or 17 or 18 or 19 or 20 or 21 or 22

25 23 and 24

**Cochrane Database of Systematic Reviews**

1 locum*.mp.

2 (contract adj2 service*).mp.

3 (staff* adj2 turnover*).mp.

4 ((job or work) adj2 satisfaction).mp.

5 ((work adj2 place*) or workplace*).mp.

6 (employment or employing).mp.

7 (hire or hiring).mp.

8 (personnel adj2 loyalty).mp.

9 (choice* or motivat* or recruit* or retention or retain* or intention* or attract* or migrat* or mobility or workforce* or disincentive* or incentive* or expectation* or staffing).mp.

10 ((labour or labor) adj2 suppl*).mp.

11 ((labour or labor) adj2 force*).mp.

12 1 or 2

13 3 or 4 or 5 or 6 or 7 or 8 or 9 or 10 or 11

14 12 and 13

**Web of Science - Core Collection**

1 TS=(locum*) OR TS=(Contract NEAR/2 Service*)

2 (TS=(loyalty) OR TS=(staff* NEAR/2 turnover*) OR TS=(job NEAR/2 satisfaction) OR TS=(work NEAR/2 satisfaction) OR TS=(work NEAR/2 place*) OR TS=(workplace*) OR TS=(employment or employing or hire or hiring) OR TS=(choice* or motivat* or recruit* or retention or retain* or intention* or attract* or migrat* or mobility or workforce* or disincentive* or incentive* or expectation* or staffing) OR TS=(labour NEAR/2 suppl*) OR TS=(labor NEAR/2 suppl*) OR TS=(labour NEAR/2 force*) OR TS=(labor NEAR/2 force*))

1 AND 2

**CINAHL**

S31 S29 AND S30

S30 S4 OR S5 OR S6 OR S7 OR S8 OR S9 OR S10 OR S11 OR S12 OR S13 OR S14 OR S15 OR S16 OR S17 OR S18 OR S19 OR S20 OR S21 OR S22 OR S23 OR S24 OR S25 OR S26 OR S27 OR S28

S29 S1 OR S2 OR S3

S28 (work N2 place*) OR (workplace*)

S27 (labor N2 force*)

S26 (labour N2 force*)

S25 (labor N2 suppl*)

S24 (labour N2 suppl*)

S23 (choice* or motivat* or recruit* or retention or retain* or intention* or attract* or migrat* or mobility or workforce* or disincentive* or incentive* or expectation* or staffing)

S22 (loyalty)

S21 (hire OR hiring)

S20 (employment OR employing)

S19 (work N2 satisfaction)

S18 (job N2 satisfaction)

S17 (staff* N2 turnover*)

S16 (MH "Personnel Selection")

S15 (MH "Employee Incentive Programs") OR (MH "Physician Incentive Plans")

S14 (MH "Personnel Loyalty")

S13 (MH "Personnel Management")

S12 (MH "Personnel Turnover")

S11 (MH "Employment")

S10 (MH "Attitude of Health Personnel") OR (MH "Physician Attitudes")

S9 (MH "Job Satisfaction")

S8 (MH "Workforce")

S7 (MH "Motivation") OR (MH "Intention")

S6 (MH "Physicians+/PF/MA")

S5 (MH "Personnel Staffing and Scheduling")

S4 (MH "Career Planning and Development")

S3 contract N2 service*

S2 (MH "Contract Services")

S1 locum*

**Appendix S2 - Mixed-methods Appraisal Tool (MMAT) quality assessment of included studies**

| Studies | Criteria from the Mixed Methods Appraisal Tool (Quantitative non-randomized studies) | | | | | |
| --- | --- | --- | --- | --- | --- | --- |
|  | 3.1 | 3.2 | 3.3 | 3.4 | 3.5 | QUALITY RATING |
| Simon & Alonzo (2004, 2008)^1,2^ | 1 | 1 | 1 | 1 | 1 | ***** |
| Rourke et al. (2003)^3^ | 1 | 1 | 1 | 0 | 1 | **** |
| Jenson et al. (2008)^4^ | 1 | 1 | 1 | 0 | 0 | *** |
| DiMeglio et al. (2018)^5^ | 0 | 1 | 1 | 0 | 1 | *** |
| McKevitt et al. (1999)^6^ | 1 | 1 | 1 | 0 | 1 | **** |

| Studies | Criteria from the Mixed Methods Appraisal Tool (Quantitative non-randomized studies (pre-post) | | | | | |
| --- | --- | --- | --- | --- | --- | --- |
|  | 3.1 | 3.2 | 3.3 | 3.4 | 3.5 | QUALITY RATING |
| Woloschuk & Tarrant (2002)^7^ | 1 | 1 | 1 | 0 | 1 | **** |

| Studies | Criteria from the Mixed Methods Appraisal Tool (Mixed-methods studies) | | | | | |
| --- | --- | --- | --- | --- | --- | --- |
|  | 5.1 | 5.2 | 5.3 | 5.4 | 5.5 | QUALITY RATING |
| Myhre et al. (2010)^8^ | 1 | 1 | 1 | 0 | 0 | *** |

| Studies | Criteria from the Mixed Methods Appraisal Tool (Qualitative studies) | | | | | |
| --- | --- | --- | --- | --- | --- | --- |
|  | 1.1 | 1.2 | 1.3 | 1.4 | 1.5 | QUALITY RATING |
| Jenson et al.  (2006)^9^ | 1 | 0 | 1 | 1 | 1 | **** |
| Rajbangshi et al. (2017)^10^ | 1 | 1 | 1 | 1 | 1 | ***** |
| Theodoulou et al. (2018)^11^ | 1 | 1 | 1 | 1 | 1 | ***** |
| Lagoo et al. (2020)^12^ | 1 | 1 | 1 | 1 | 1 | ***** |

**References**

1. Simon AB, Alonzo AA. The demography, career pattern, and motivation of Locum tenens physicians in the United States. Journal of Healthcare Management. 2004;49(6):363–75.

2. Alonzo AA, Simon AB. Have stethoscope, will travel: contingent employment among physician health care providers in the United States. WORK EMPLOYMENT AND SOCIETY. 2008;22(4):635–54.

3. Rourke JTB, Incitti F, Rourke LL, Kennard MA. Keeping family physicians in rural practice - Solutions favoured by rural physicians and family medicine residents. Canadian Family Physician. 2003;49:1142–9.

4. Jenson C, Reid F, Rowlands G. Locum and salaried general practitioners: an exploratory study of recruitment, morale, professional development and clinical governance. Education for Primary Care [Internet]. 2008;19(3):285–302. Available from: <https://doi.org/10.1080/14739879.2008.11493685>

5. DiMeglio M, Furey W, Laudanski K. Content analysis of locum tenens recruitment emails for anesthesiologists. BMC Health Serv Res [Internet]. 2018;18(1):N.PAG-N.PAG. Available from: <https://doi.org/10.1186/s12913-018-3758-6>

6. McKevitt C, Morgan M, Hudson M. Locum doctors in general practice: motivation and experiences. British Journal of General Practice [Internet]. 1999;49(444):519–21.

7. Woloschuk W, Tarrant M. Does a rural educational experience influence students’ likelihood of rural practice? Impact of student background and gender. Med Educ. 2002;36(3):241–7.

8. Myhre DL, Konkin J, Woloschuk W, Szafran O, Hansen C, Crutcher R, et al. Locum practice by recent family medicine graduates. Canadian Family Physician [Internet]. 2010;56(5):e183-90.

9. Jenson CM, Hutchins AJ, Rowlands G. Is small-group education the key to retention of sessional GPs? Education for primary care. 2006;17(3):218–26.

10. Rajbangshi PR, Nambiar D, Choudhury N, Rao KD. Rural recruitment and retention of health workers across cadres and types of contract in north-east India: A qualitative study. WHO South East Asia J Public Health. 2017;6(2):51–9.

11. Theodoulou I, Reddy AM, Wong J. Is innovative workforce planning software the solution to NHS staffing and cost crisis? An exploration of the locum industry. BMC Health Serv Res [Internet]. 2018;18:1. Available from: <https://doi.org/10.1186/s12913-018-2989-x>

12. Lagoo J, Berry W, Henrich N, Gawande A, Sato L, Haas S. Safely Practicing in a New Environment: A Qualitative Study to Inform Physician Onboarding Practices. Jt Comm J Qual Patient Saf [Internet]. 2020;46(6):314–20. Available from: <https://doi.org/10.1016/j.jcjq.2020.03.002>

**Appendix S3 - Grey Literature Search Strategy, Data Extraction, and Evaluation**

Between June 12th and July 16th, 2023 we performed a systematic search of grey literature. Databases included search engines such as google, grey literature repositories such as OpenGrey, and Public Health Ontario. Examples of the search terms and headings applied include “Locum”, “Contract”, “Temporary”, and “Locum Physician”. Limitations were applied to the searches. Articles beyond 1990, and for certain databases, any piece of information passed 150 or 250 search results were not analyzed. For full search histories please see Supplemental Content below. This search strategy was developed in collaboration with the librarian on the team who completed each database search.

Grey literature pieces were stored in Mendeley and imported into Covidence systematic review software for further screening. Same exclusion criteria was applied as was in the scholarly search. One reviewer independently screened (see Figure S1 below), extracted data and evaluated grey literature for quality. Screening took place using Covidence, whereas data extraction and evaluation took place on google sheets. The evaluation tool used was the AACODS Checklist for appraisal of grey literature.^1^ See supplemental file below detailing our evaluation and extraction “Data Extraction and Evaluation of Grey Literature”.


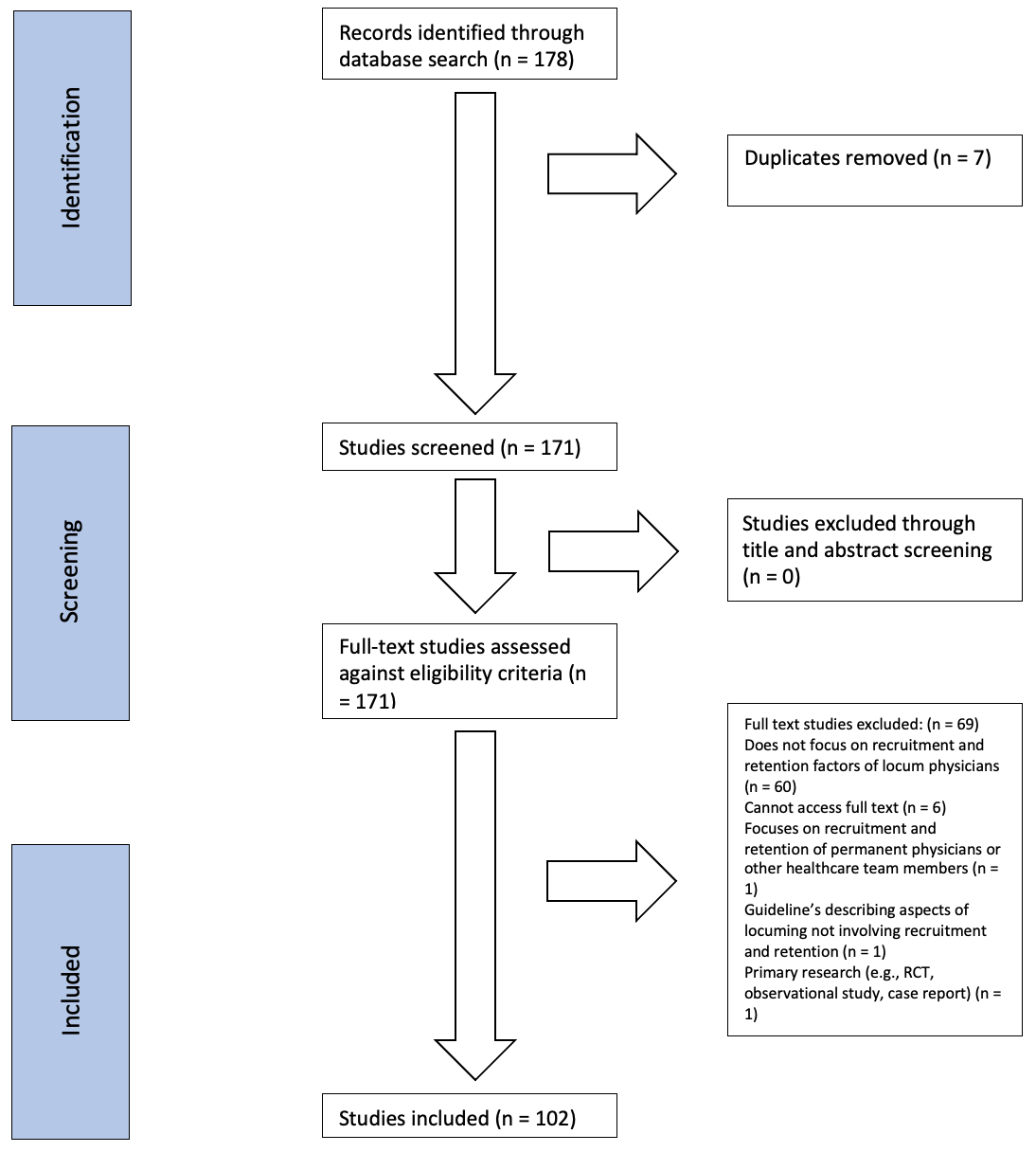


**Figure S1**: PRISMA flow diagram detailing the selection process of grey literature.

**REFERENCES**

1. Tyndall J. AACODS Checklist. Flinders University. 2010. Available from: <https://www.library.sydney.edu.au/research/systematic-review/downloads/AACODS_Checklist.pdf>

**Supplemental:**

Relevant authorities, domains, organizations, websites after search:

- Keywords:
  - Locum
  - Locum tenens
  - Locum AND physician
  - Locum physicians
  - Locum recruitment and retention
  - Locum physician recruitment and retention
  - Locum physician recruitment
  - Locum physician retention
  - Temporary physician recruitment and retention
  - Temporary physician recruitment
  - Temporary physician retention
  - Contract physician recruitment and retention
  - Contract physician recruitment
  - Contract physician retention
  - Temporary physician
  - Temporary physicians
  - Temporary doctor
  - Temporary doctors
  - Contract physician
  - Contract physicians
  - Contract doctor
  - Contract doctors
- Domains:
  - .ca
  - .gov
  - .gov.on.ca
  - .com
  - .edu
  - .net
  - Country specific (e.g., .uk, .in, .ca, .com)

**Search limits:**

By country and databases: Canada, UK, USA, India, Australia

- CANADA
  - In depth search (>10 websites, > 5 Grey lit databases, 1 google.ca search)
- UK
  - Limit to:
    - 5 websites
    - 1 google.uk search
- USA
  - Limit to:
    - 5 websites
    - 1 google.com search
- India
  - Limit to:
    - 5 websites
    - 1 google.in search
- Australia
  - Limit to:
    - 6 websites
    - 1 google.au search

By date: 1990 onward

- Compare 2005 limit to 1990 limit
  - If similar in articles retrieved (number and type), limit to more recent time (2005)

By language: English

**Website Searching Documentation (Strategy 1)**

| Date | Organization name & website URL | Search strategy(s)/ words searched including (if applicable) how items were selected. | # items retrieved/ search results | # of items screened (uploaded to citation management software) |
| --- | --- | --- | --- | --- |
| 12Jun2023 | HealthForceOntario; <https://www.healthforceontario.ca/en/Home/All_Programs> | Used Advanced Google site/domain search  Search strategies:  Search region limited to: Canada  1) This exact word or phrase: “locum”; any format (e.g., pdf, .xlsx, .doc)  Limited to: English language documents; Canada; without the words: nurse, job, ad, ads, advertisement, midwife, pharmacist  2) All these word(s): “locum”; any format (e.g., pdf, .xlsx, .doc)  Limited to: English language documents; Canada; without the words: nurse, job, ad, ads, advertisement, midwife, pharmacist  3) All these word(s): “locum”; any format (e.g., pdf, .xlsx, .doc)  Selection:  All items retrieved with each search were reviewed for relevance by 1 reviewer.  Items were selected by scanning the first 250 results from each search. | 1) 40  2) 29  3) 66 | 1) 2  2) 0  3) 4 |
| 12Jun2023 | The College of Family Physicians of Canada; cfpc.ca | Used Advanced Google site/domain search  Search strategies:  Search region limited to: Canada  1) This exact word or phrase: “locum”; any format (e.g., pdf, .xlsx, .doc)  Limited to: English language documents; Canada; without the words: nurse, job, ad, ads, advertisement, midwife, pharmacist  2) All these word(s): “locum”; any format (e.g., pdf, .xlsx, .doc)  Limited to: English language documents; Canada; without the words: nurse, job, ad, ads, advertisement, midwife, pharmacist  Selection:  All items retrieved with each search were reviewed for relevance by 1 reviewer.  Items were selected by scanning the first 250 results from each search. | 1) 282  2) 437 | 1) 2  2) 6 |
| 13Jun2023 | Ontario College of Family Physicians; ontariofamilyphysicians.ca | Used Advanced Google site/domain search  Search strategies:  Search region limited to: Canada  1) This exact word or phrase: “locum”; any format (e.g., pdf, .xlsx, .doc)  Limited to: English language documents; Canada; without the words: nurse, job, ad, ads, advertisement, midwife, pharmacist  2) All these word(s): “locum”; any format (e.g., pdf, .xlsx, .doc)  Limited to: English language documents; Canada; without the words: nurse, job, ad, ads, advertisement, midwife, pharmacist  3) All these word(s): “locum”; any format (e.g., pdf, .xlsx, .doc)  Selection:  All items retrieved with each search were reviewed for relevance by 1 reviewer.  Items were selected by scanning the first 250 results from each search. | 1) 6  2) 37  3) 35 | 1) 0  2) 1  3) 0 |
| 13Jun2023 | CBC.ca; cbc.ca | Used Advanced Google site/domain search  Search strategies:  Search region limited to: Canada  1) This exact word or phrase: “locum”; any format (e.g., pdf, .xlsx, .doc)  Limited to: English language documents; Canada; without the words: nurse, job, ad, ads, advertisement, midwife, pharmacist  2) All these word(s): “locum”; any format (e.g., pdf, .xlsx, .doc)  Limited to: English language documents; Canada; without the words: nurse, job, ad, ads, advertisement, midwife, pharmacist  Selection:  All items retrieved with each search were reviewed for relevance by 1 reviewer.  Items were selected by scanning the first 250 results from each search. | 1) 9  2) 199 | 1) 0  2) 3 |
| 13Jun2023 | Canada.ca; Canada.ca | Used Advanced Google site/domain search  Search strategies:  Search region limited to: Canada  1) This exact word or phrase: “locum”; any format (e.g., pdf, .xlsx, .doc)  Limited to: English language documents; Canada; without the words: nurse, job, ad, ads, advertisement, midwife, pharmacist  2) All these word(s): “locum”; any format (e.g., pdf, .xlsx, .doc)  Limited to: English language documents; Canada; without the words: nurse, job, ad, ads, advertisement, midwife, pharmacist  3) All these word(s): “locum”; any format (e.g., pdf, .xlsx, .doc)  Selection:  All items retrieved with each search were reviewed for relevance by 1 reviewer.  Items were selected by scanning the first 250 results from each search. | 1) 1  2) 2  3) 166 | 1) 0  2) 0  3) 1 |
| 13Jun2023 | Public Health Ontario; publichealthontario.ca | Used Advanced Google site/domain search  Search strategies:  Search region limited to: Canada  1) This exact word or phrase: “locum”; any format (e.g., pdf, .xlsx, .doc)  Limited to: English language documents; Canada; without the words: nurse, job, ad, ads, advertisement, midwife, pharmacist  2) All these word(s): “locum”; any format (e.g., pdf, .xlsx, .doc)  Limited to: English language documents; Canada; without the words: nurse, job, ad, ads, advertisement, midwife, pharmacist  3) All these word(s): “locum”; any format (e.g., pdf, .xlsx, .doc)  Selection:  All items retrieved with each search were reviewed for relevance by 1 reviewer.  Items were selected by scanning the first 250 results from each search. | 1) 0  2) 0  3) 1 | 1) 0  2) 0  3) 0 |
| 13Jun2023 | Society of Rural Physicians of Canada (SRPC); srpc.ca | Used Advanced Google site/domain search  Search strategies:  Search region limited to: Canada  1) This exact word or phrase: “locum”; any format (e.g., pdf, .xlsx, .doc)  Limited to: English language documents; Canada; without the words: nurse, job, ad, ads, advertisement, midwife, pharmacist  2) All these word(s): “locum”; any format (e.g., pdf, .xlsx, .doc)  Limited to: English language documents; Canada; without the words: nurse, job, ad, ads, advertisement, midwife, pharmacist  Selection:  All items retrieved with each search were reviewed for relevance by 1 reviewer.  Items were selected by scanning the first 250 results from each search. | 1) 102  2) 168 | 1) 5  2) 5 |
| 13Jun2023 | Canadian Medical Association; cma.ca | Used Advanced Google site/domain search  Search strategies:  Search region limited to: Canada  1) This exact word or phrase: “locum”; any format (e.g., pdf, .xlsx, .doc)  Limited to: English language documents; Canada; without the words: nurse, job, ad, ads, advertisement, midwife, pharmacist  2) All these word(s): “locum”; any format (e.g., pdf, .xlsx, .doc)  Limited to: English language documents; Canada; without the words: nurse, job, ad, ads, advertisement, midwife, pharmacist  3) All these word(s): “locum”; any format (e.g., pdf, .xlsx, .doc)  Selection:  All items retrieved with each search were reviewed for relevance by 1 reviewer.  Items were selected by scanning the first 250 results from each search. | 1) 88  2) 54  3) 142 | 1) 3  2) 0  3) 4 |
| 6Jul2023 | Healthy Debate; [healthydebate.ca](https://healthydebate.ca/) | Used Advanced Google site/domain search  Search strategies:  Search region limited to: Canada  1) This exact word or phrase: “locum”; any format (e.g., pdf, .xlsx, .doc)  Limited to: English language documents; Canada; without the words: nurse, job, ad, ads, advertisement, midwife, pharmacist  2) All these word(s): “locum”; any format (e.g., pdf, .xlsx, .doc)  Limited to: English language documents; Canada; without the words: nurse, job, ad, ads, advertisement, midwife, pharmacist  3) All these word(s): “locum”; any format (e.g., pdf, .xlsx, .doc)  Selection:  All items retrieved with each search were reviewed for relevance by 1 reviewer.  Items were selected by scanning the first 250 results from each search. | 1) 6  2) 6  3) 32 | 1) 1  2) 0  3) 3 |
| 7Jul2023 | The Government of British Columbia; gov.bc.ca | Used Advanced Google site/domain search  Search strategies:  Search region limited to: Canada  1) This exact word or phrase: “locum”; any format (e.g., pdf, .xlsx, .doc)  Limited to: English language documents; Canada; without the words: nurse, job, ad, ads, advertisement, midwife, pharmacist  2) All these word(s): “locum”; any format (e.g., pdf, .xlsx, .doc)  Limited to: English language documents; Canada; without the words: nurse, job, ad, ads, advertisement, midwife, pharmacist  3) All these word(s): “locum”; any format (e.g., pdf, .xlsx, .doc)  Selection:  All items retrieved with each search were reviewed for relevance by 1 reviewer.  Items were selected by scanning the first 250 results from each search. | 1) 183  2) 170  3) 597 | 1) 1  2) 0  3) 0 |
| 10Jul2023 | Rural Coordination Centre of British Columbia; rccbc.ca | Used Advanced Google site/domain search  Search strategies:  Search region limited to: Canada  1) This exact word or phrase: “locum”; any format (e.g., pdf, .xlsx, .doc)  Limited to: English language documents; Canada; without the words: nurse, job, ad, ads, advertisement, midwife, pharmacist  2) All these word(s): “locum”; any format (e.g., pdf, .xlsx, .doc)  Limited to: English language documents; Canada; without the words: nurse, job, ad, ads, advertisement, midwife, pharmacist  3) All these word(s): “locum”; any format (e.g., pdf, .xlsx, .doc)  Selection:  All items retrieved with each search were reviewed for relevance by 1 reviewer.  Items were selected by scanning the first 250 results from each search. | 1) 37  2) 78  3) 57 | 1) 4  2) 1  3) 2 |
| 10Jul2023 | Alberta Medical Association; albertadoctors.org | Used Advanced Google site/domain search  Search strategies:  Search region limited to: Canada  1) This exact word or phrase: “locum”; any format (e.g., pdf, .xlsx, .doc)  Limited to: English language documents; Canada; without the words: nurse, job, ad, ads, advertisement, midwife, pharmacist  2) All these word(s): “locum”; any format (e.g., pdf, .xlsx, .doc)  Limited to: English language documents; Canada; without the words: nurse, job, ad, ads, advertisement, midwife, pharmacist  3) All these word(s): “locum”; any format (e.g., pdf, .xlsx, .doc)  Selection:  All items retrieved with each search were reviewed for relevance by 1 reviewer.  Items were selected by scanning the first 250 results from each search. | 1) 661  2) 1240  3) 930 | 1) 0  2) 0  3) 0 |
| 10Jul2023 | Gov’t of Saskatchewan; saskatchewan.ca | Used Advanced Google site/domain search  Search strategies:  Search region limited to: Canada  1) This exact word or phrase: “locum”; any format (e.g., pdf, .xlsx, .doc)  Limited to: English language documents; Canada; without the words: nurse, job, ad, ads, advertisement, midwife, pharmacist  2) All these word(s): “locum”; any format (e.g., pdf, .xlsx, .doc)  Limited to: English language documents; Canada; without the words: nurse, job, ad, ads, advertisement, midwife, pharmacist  3) All these word(s): “locum”; any format (e.g., pdf, .xlsx, .doc)  Selection:  All items retrieved with each search were reviewed for relevance by 1 reviewer.  Items were selected by scanning the first 250 results from each search. | 1) 23  2) 23  3) 68 | 1) 1  2) 0  3) 0 |
| 10Jul2023 | Manitoba Locum Tenens Program; manitobalocum.ca | Used Advanced Google site/domain search  Search strategies:  Search region limited to: Canada  1) This exact word or phrase: “locum”; any format (e.g., pdf, .xlsx, .doc)  Limited to: English language documents; Canada; without the words: nurse, job, ad, ads, advertisement, midwife, pharmacist  2) All these word(s): “locum”; any format (e.g., pdf, .xlsx, .doc)  Limited to: English language documents; Canada; without the words: nurse, job, ad, ads, advertisement, midwife, pharmacist  3) All these word(s): “locum”; any format (e.g., pdf, .xlsx, .doc)  Selection:  All items retrieved with each search were reviewed for relevance by 1 reviewer.  Items were selected by scanning the first 250 results from each search. | 1) 6  2) 6  3) 39 | 1) 1  2) 0  3) 0 |
| 10Jul2023 | Province of Manitoba; gov.mb.ca | Used Advanced Google site/domain search  Search strategies:  Search region limited to: Canada  1) This exact word or phrase: “locum”; any format (e.g., pdf, .xlsx, .doc)  Limited to: English language documents; Canada; without the words: nurse, job, ad, ads, advertisement, midwife, pharmacist  2) All these word(s): “locum”; any format (e.g., pdf, .xlsx, .doc)  Limited to: English language documents; Canada; without the words: nurse, job, ad, ads, advertisement, midwife, pharmacist  3) All these word(s): “locum”; any format (e.g., pdf, .xlsx, .doc)  Selection:  All items retrieved with each search were reviewed for relevance by 1 reviewer.  Items were selected by scanning the first 250 results from each search. | 1) 23  2) 23  3) 230 | 1) 0  2) 0  3) 0 |
| 10Jul2023 | Yukon Medical Association; yukondoctors.ca | Used Advanced Google site/domain search  Search strategies:  Search region limited to: Canada  1) This exact word or phrase: “locum”; any format (e.g., pdf, .xlsx, .doc)  Limited to: English language documents; Canada; without the words: nurse, job, ad, ads, advertisement, midwife, pharmacist  2) All these word(s): “locum”; any format (e.g., pdf, .xlsx, .doc)  Limited to: English language documents; Canada; without the words: nurse, job, ad, ads, advertisement, midwife, pharmacist  3) All these word(s): “locum”; any format (e.g., pdf, .xlsx, .doc)  Selection:  All items retrieved with each search were reviewed for relevance by 1 reviewer.  Items were selected by scanning the first 250 results from each search. | 1) 6  2) 6  3) 31 | 1) 1  2) 0  3) 0 |
| 12Jul2023 | Yukon Gov’t; yukon.ca | Used Advanced Google site/domain search  Search strategies:  Search region limited to: Canada  1) This exact word or phrase: “locum”; any format (e.g., pdf, .xlsx, .doc)  Limited to: English language documents; Canada; without the words: nurse, job, ad, ads, advertisement, midwife, pharmacist  2) All these word(s): “locum”; any format (e.g., pdf, .xlsx, .doc)  Limited to: English language documents; Canada; without the words: nurse, job, ad, ads, advertisement, midwife, pharmacist  3) All these word(s): “locum”; any format (e.g., pdf, .xlsx, .doc)  Selection:  All items retrieved with each search were reviewed for relevance by 1 reviewer.  Items were selected by scanning the first 250 results from each search. | 1) 8  2) 9  3) 35 | 1) 0  2) 0  3) 2 |
| 12Jul2023 | Practice NWT; practicenwt.ca | Used Advanced Google site/domain search  Search strategies:  Search region limited to: Canada  1) This exact word or phrase: “locum”; any format (e.g., pdf, .xlsx, .doc)  Limited to: English language documents; Canada; without the words: nurse, job, ad, ads, advertisement, midwife, pharmacist  2) All these word(s): “locum”; any format (e.g., pdf, .xlsx, .doc)  Limited to: English language documents; Canada; without the words: nurse, job, ad, ads, advertisement, midwife, pharmacist  3) All these word(s): “locum”; any format (e.g., pdf, .xlsx, .doc)  Selection:  All items retrieved with each search were reviewed for relevance by 1 reviewer.  Items were selected by scanning the first 250 results from each search. | 1) 58  2) 76  3) 130 | 1) 3  2) 0  3) 3 |
| 12Jul2023 | NWT Gov’t; gov.nt.ca | Used Advanced Google site/domain search  Search strategies:  Search region limited to: Canada  1) This exact word or phrase: “locum”; any format (e.g., pdf, .xlsx, .doc)  Limited to: English language documents; Canada; without the words: nurse, job, ad, ads, advertisement, midwife, pharmacist  2) All these word(s): “locum”; any format (e.g., pdf, .xlsx, .doc)  Limited to: English language documents; Canada; without the words: nurse, job, ad, ads, advertisement, midwife, pharmacist  3) All these word(s): “locum”; any format (e.g., pdf, .xlsx, .doc)  Selection:  All items retrieved with each search were reviewed for relevance by 1 reviewer.  Items were selected by scanning the first 250 results from each search. | 1) 45  2) 47  3) 468 | 1) 0  2) 0  3) 2 |
| 12Jul2023 | Nunavut Gov’t; gov.nu.ca | Used Advanced Google site/domain search  Search strategies:  Search region limited to: Canada  1) This exact word or phrase: “locum”; any format (e.g., pdf, .xlsx, .doc)  Limited to: English language documents; Canada; without the words: nurse, job, ad, ads, advertisement, midwife, pharmacist  2) All these word(s): “locum”; any format (e.g., pdf, .xlsx, .doc)  Limited to: English language documents; Canada; without the words: nurse, job, ad, ads, advertisement, midwife, pharmacist  3) All these word(s): “locum”; any format (e.g., pdf, .xlsx, .doc)  Selection:  All items retrieved with each search were reviewed for relevance by 1 reviewer.  Items were selected by scanning the first 250 results from each search. | 1) 1  2) 2  3) 55 | 1) 0  2) 0  3) 0 |
| 12Jul2023 | Gov’t of Newfoundland; gov.nl.ca | Used Advanced Google site/domain search  Search strategies:  Search region limited to: Canada  1) This exact word or phrase: “locum”; any format (e.g., pdf, .xlsx, .doc)  Limited to: English language documents; Canada; without the words: nurse, job, ad, ads, advertisement, midwife, pharmacist  2) All these word(s): “locum”; any format (e.g., pdf, .xlsx, .doc)  Limited to: English language documents; Canada; without the words: nurse, job, ad, ads, advertisement, midwife, pharmacist  3) All these word(s): “locum”; any format (e.g., pdf, .xlsx, .doc)  Selection:  All items retrieved with each search were reviewed for relevance by 1 reviewer.  Items were selected by scanning the first 250 results from each search. | 1) 68  2) 74  3) 314 | 1) 1  2) 0  3) 1 |
| 12Jul2023 | Newfoundland and Labrador Medical Association; nlma.nl.ca | Used Advanced Google site/domain search  Search strategies:  Search region limited to: Canada  1) This exact word or phrase: “locum”; any format (e.g., pdf, .xlsx, .doc)  Limited to: English language documents; Canada; without the words: nurse, job, ad, ads, advertisement, midwife, pharmacist  2) All these word(s): “locum”; any format (e.g., pdf, .xlsx, .doc)  Limited to: English language documents; Canada; without the words: nurse, job, ad, ads, advertisement, midwife, pharmacist  3) All these word(s): “locum”; any format (e.g., pdf, .xlsx, .doc)  Selection:  All items retrieved with each search were reviewed for relevance by 1 reviewer.  Items were selected by scanning the first 250 results from each search. | 1) 38  2) 67  3) 134 | 1) 3  2) 0  3) 2 |
| 13Jul2023 | Doctors of Nova Scotia; doctorsns.com | Used Advanced Google site/domain search  Search strategies:  Search region limited to: Canada  1) This exact word or phrase: “locum”; any format (e.g., pdf, .xlsx, .doc)  Limited to: English language documents; Canada; without the words: nurse, job, ad, ads, advertisement, midwife, pharmacist  2) All these word(s): “locum”; any format (e.g., pdf, .xlsx, .doc)  Limited to: English language documents; Canada; without the words: nurse, job, ad, ads, advertisement, midwife, pharmacist  3) All these word(s): “locum”; any format (e.g., pdf, .xlsx, .doc)  Selection:  All items retrieved with each search were reviewed for relevance by 1 reviewer.  Items were selected by scanning the first 250 results from each search. | 1) 0  2) 0  3) 428 | 1) 0  2) 0  3) 1 |
| 16Jun2023 | NHS England; <https://www.england.nhs.uk/> | Used Advanced Google site/domain search  Search strategies:  Search region limited to: United Kingdom  1) This exact word or phrase: “locum”; any format (e.g., pdf, .xlsx, .doc)  Limited to: English language documents; United Kingdom; without the words: nurse, job, ad, ads, advertisement, midwife, pharmacist  2) All these word(s): “locum”; any format (e.g., pdf, .xlsx, .doc)  Limited to: English language documents; United Kingdom; without the words: nurse, job, ad, ads, advertisement, midwife, pharmacist  Selection:  All items retrieved with each search were reviewed for relevance by 1 reviewer.  Items were selected by scanning the first 150 results from each search. | 1) 259  2) 398 | 1) 6  2) 0 |
| 16Jun2023 | The Health Foundation; <https://www.health.org.uk/> | Used Advanced Google site/domain search  Search strategies:  Search region limited to: United Kingdom  1) This exact word or phrase: “locum”; any format (e.g., pdf, .xlsx, .doc)  Limited to: English language documents; United Kingdom; without the words: nurse, job, ad, ads, advertisement, midwife, pharmacist  2) All these word(s): “locum”; any format (e.g., pdf, .xlsx, .doc)  Limited to: English language documents; United Kingdom; without the words: nurse, job, ad, ads, advertisement, midwife, pharmacist  3) All these word(s): “locum”; any format (e.g., pdf, .xlsx, .doc)  4) This exact word or phrase: “sessional GP”; any format (e.g., pdf, .xlsx, .doc)  Limited to: English language documents; United Kingdom; without the words: nurse, job, ad, ads, advertisement, midwife, pharmacist  5) This exact word or phrase: “sessional GP”; any format (e.g., pdf, .xlsx, .doc)  6) All these words(s): “sessional GP”; any format (e.g., pdf, .xlsx, .doc)  Selection:  All items retrieved with each search were reviewed for relevance by 1 reviewer.  Items were selected by scanning the first 150 results from each search. | 1) 10  2) 21  3) 104  4) 0  5) 40  6) 43 | 1) 0  2) 0  3) 0  4) 0  5) 0  6) 0 |
| 17Jun2023 | Royal Society for Public Health; <https://www.rsph.org.uk/> | Used Advanced Google site/domain search  Search strategies:  Search region limited to: United Kingdom  1) This exact word or phrase: “locum”; any format (e.g., pdf, .xlsx, .doc)  Limited to: English language documents; United Kingdom; without the words: nurse, job, ad, ads, advertisement, midwife, pharmacist  2) All these word(s): “locum”; any format (e.g., pdf, .xlsx, .doc)  Limited to: English language documents; United Kingdom; without the words: nurse, job, ad, ads, advertisement, midwife, pharmacist  3) All these word(s): “locum”; any format (e.g., pdf, .xlsx, .doc)  4) This exact word or phrase: “sessional GP”; any format (e.g., pdf, .xlsx, .doc)  Limited to: English language documents; United Kingdom; without the words: nurse, job, ad, ads, advertisement, midwife, pharmacist  5) This exact word or phrase: “sessional GP”; any format (e.g., pdf, .xlsx, .doc)  6) All these words(s): “sessional GP”; any format (e.g., pdf, .xlsx, .doc)  Selection:  All items retrieved with each search were reviewed for relevance by 1 reviewer.  Items were selected by scanning the first 150 results from each search. | 1) 0  2) 0  3) 2  4) 0  5) 0  6) 0 | 1) 0  2) 0  3) 0  4) 0  5) 0  6) 0 |
| 17Jun2023 | Public Health England; <https://www.gov.uk/government/organisations/public-health-england> | Used Advanced Google site/domain search  Search strategies:  Search region limited to: United Kingdom  1) This exact word or phrase: “locum”; any format (e.g., pdf, .xlsx, .doc)  Limited to: English language documents; United Kingdom; without the words: nurse, job, ad, ads, advertisement, midwife, pharmacist  2) All these word(s): “locum”; any format (e.g., pdf, .xlsx, .doc)  Limited to: English language documents; United Kingdom; without the words: nurse, job, ad, ads, advertisement, midwife, pharmacist  3) All these word(s): “locum”; any format (e.g., pdf, .xlsx, .doc)  4) This exact word or phrase: “sessional GP”; any format (e.g., pdf, .xlsx, .doc)  Limited to: English language documents; United Kingdom; without the words: nurse, job, ad, ads, advertisement, midwife, pharmacist  5) This exact word or phrase: “sessional GP”; any format (e.g., pdf, .xlsx, .doc)  6) All these words(s): “sessional GP”; any format (e.g., pdf, .xlsx, .doc)  Selection:  All items retrieved with each search were reviewed for relevance by 1 reviewer.  Items were selected by scanning the first 150 results from each search. | 1) 0  2) 0  3) 0  4) 0  5) 0  6) 0 | 1) 0  2) 0  3) 0  4) 0  5) 0  6) 0 |
| 6Jul2023 | The University of Manchester; manchester.ac.uk | Used Advanced Google site/domain search  Search strategies:  Search region limited to: United Kingdom  1) This exact word or phrase: “locum”; any format (e.g., pdf, .xlsx, .doc)  Limited to: English language documents; United Kingdom; without the words: nurse, job, ad, ads, advertisement, midwife, pharmacist  2) All these word(s): “locum”; any format (e.g., pdf, .xlsx, .doc)  Limited to: English language documents; United Kingdom; without the words: nurse, job, ad, ads, advertisement, midwife, pharmacist  3) All these word(s): “locum”; any format (e.g., pdf, .xlsx, .doc)  Selection:  All items retrieved with each search were reviewed for relevance by 1 reviewer.  Items were selected by scanning the first 150 results from each search. | 1) 120  2) 153  3) 614 | 1) 0  2) 0  3) 0 |
| 19Jun2023 | U.S Department of Health and Human Services; <https://www.hhs.gov/> | Used Advanced Google site/domain search  Search strategies:  Search region limited to: United States  1) This exact word or phrase: “locum”; any format (e.g., pdf, .xlsx, .doc)  Limited to: English language documents; United States; without the words: nurse, job, ad, ads, advertisement, midwife, pharmacist  2) All these word(s): “locum”; any format (e.g., pdf, .xlsx, .doc)  Limited to: English language documents; United States; without the words: nurse, job, ad, ads, advertisement, midwife, pharmacist  3) All these word(s): “locum”; any format (e.g., pdf, .xlsx, .doc)  Selection:  All items retrieved with each search were reviewed for relevance by 1 reviewer.  Items were selected by scanning the first 150 results from each search. | 1) 31  2) 31  3) 45 | 1) 0  2) 0  3) 0 |
| 19Jun2023 | Rural Health Information Hub; <https://www.ruralhealthinfo.org/> | Used Advanced Google site/domain search  Search strategies:  Search region limited to: United States  1) This exact word or phrase: “locum”; any format (e.g., pdf, .xlsx, .doc)  Limited to: English language documents; United States; without the words: nurse, job, ad, ads, advertisement, midwife, pharmacist  2) All these word(s): “locum”; any format (e.g., pdf, .xlsx, .doc)  Limited to: English language documents; United States; without the words: nurse, job, ad, ads, advertisement, midwife, pharmacist  3) All these word(s): “locum”; any format (e.g., pdf, .xlsx, .doc)  Selection:  All items retrieved with each search were reviewed for relevance by 1 reviewer.  Items were selected by scanning the first 150 results from each search. | 1) 2  2) 2  3) 38 | 1) 1  2) 0  3) 1 |
| 19Jun2023 | National Rural Health Association; <https://www.ruralhealth.us/> | Used Advanced Google site/domain search  Search strategies:  Search region limited to: United States  1) This exact word or phrase: “locum”; any format (e.g., pdf, .xlsx, .doc)  Limited to: English language documents; United States; without the words: nurse, job, ad, ads, advertisement, midwife, pharmacist  2) All these word(s): “locum”; any format (e.g., pdf, .xlsx, .doc)  Limited to: English language documents; United States; without the words: nurse, job, ad, ads, advertisement, midwife, pharmacist  3) All these word(s): “locum”; any format (e.g., pdf, .xlsx, .doc)  Selection:  All items retrieved with each search were reviewed for relevance by 1 reviewer.  Items were selected by scanning the first 150 results from each search. | 1) 1  2) 1  3) 37 | 1) 0  2) 0  3) 0 |
| 19Jun2023 | Centre for Medicare & Medicaid Services; <https://www.cms.gov/> | Used Advanced Google site/domain search  Search strategies:  Search region limited to: United States  1) This exact word or phrase: “locum”; any format (e.g., pdf, .xlsx, .doc)  Limited to: English language documents; United States; without the words: nurse, job, ad, ads, advertisement, midwife, pharmacist  2) All these word(s): “locum”; any format (e.g., pdf, .xlsx, .doc)  Limited to: English language documents; United States; without the words: nurse, job, ad, ads, advertisement, midwife, pharmacist  3) All these word(s): “locum”; any format (e.g., pdf, .xlsx, .doc)  Selection:  All items retrieved with each search were reviewed for relevance by 1 reviewer.  Items were selected by scanning the first 150 results from each search. | 1) 37  2) 39  3) 327 | 1) 0  2) 0  3) 0 |
| 19Jun2023 | Health Resources and Services Administration; <https://www.hrsa.gov/> | Used Advanced Google site/domain search  Search strategies:  Search region limited to: United States  1) This exact word or phrase: “locum”; any format (e.g., pdf, .xlsx, .doc)  Limited to: English language documents; United States; without the words: nurse, job, ad, ads, advertisement, midwife, pharmacist  2) All these word(s): “locum”; any format (e.g., pdf, .xlsx, .doc)  Limited to: English language documents; United States; without the words: nurse, job, ad, ads, advertisement, midwife, pharmacist  3) All these word(s): “locum”; any format (e.g., pdf, .xlsx, .doc)  Selection:  All items retrieved with each search were reviewed for relevance by 1 reviewer.  Items were selected by scanning the first 150 results from each search. | 1) 0  2) 1  3) 9 | 1) 0  2) 0  3) 0 |
| 21Jun2023 | Ministry of Health and Family Welfare; <https://main.mohfw.gov.in/> | Used Advanced Google site/domain search  Search strategies:  Search region limited to: India  1) This exact word or phrase: “locum”; any format (e.g., pdf, .xlsx, .doc)  Limited to: English language documents; India; without the words: nurse, job, ad, ads, advertisement, midwife, pharmacist  2) All these word(s): “locum”; any format (e.g., pdf, .xlsx, .doc)  Limited to: English language documents; India; without the words: nurse, job, ad, ads, advertisement, midwife, pharmacist  3) All these word(s): “locum”; any format (e.g., pdf, .xlsx, .doc)  4) This exact word or phrase: “contract doctor”; any format (e.g., pdf, .xlsx, .doc)  Limited to: English language documents; India  Selection:  All items retrieved with each search were reviewed for relevance by 1 reviewer.  Items were selected by scanning the first 150 results from each search. | 1) 0  2) 0  3) 0  4) 556 | 1) 0  2) 0  3) 0  4) 0 |
| 21Jun2023 | National Health Mission; <https://nhm.gov.in/> | Used Advanced Google site/domain search  Search strategies:  Search region limited to: India  1) This exact word or phrase: “locum”; any format (e.g., pdf, .xlsx, .doc)  Limited to: English language documents; India; without the words: nurse, job, ad, ads, advertisement, midwife, pharmacist  2) All these word(s): “locum”; any format (e.g., pdf, .xlsx, .doc)  Limited to: English language documents; India; without the words: nurse, job, ad, ads, advertisement, midwife, pharmacist  3) All these word(s): “locum”; any format (e.g., pdf, .xlsx, .doc)  4) This exact word or phrase: “contract doctor”; any format (e.g., pdf, .xlsx, .doc)  Limited to: English language documents; India  Selection:  All items retrieved with each search were reviewed for relevance by 1 reviewer.  Items were selected by scanning the first 150 results from each search. | 1) 0  2) 0  3) 0  4) 511 | 1) 0  2) 0  3) 0  4) 2 |
| 21Jun2023 | Government of India - MyGov; [www.mygov.in](http://www.mygov.in) | Used Advanced Google site/domain search  Search strategies:  Search region limited to: India  1) This exact word or phrase: “locum”; any format (e.g., pdf, .xlsx, .doc)  Limited to: English language documents; India; without the words: nurse, job, ad, ads, advertisement, midwife, pharmacist  2) All these word(s): “locum”; any format (e.g., pdf, .xlsx, .doc)  Limited to: English language documents; India; without the words: nurse, job, ad, ads, advertisement, midwife, pharmacist  3) All these word(s): “locum”; any format (e.g., pdf, .xlsx, .doc)  4) This exact word or phrase: “contract doctor”; any format (e.g., pdf, .xlsx, .doc)  Limited to: English language documents; India  Selection:  All items retrieved with each search were reviewed for relevance by 1 reviewer.  Items were selected by scanning the first 150 results from each search. | 1) 0  2) 0  3) 0  4) 495 | 1) 0  2) 0  3) 0  4) 0 |
| 21Jun2023 | Public Health Foundation of India; phfi.org | Used Advanced Google site/domain search  Search strategies:  Search region limited to: India  1) This exact word or phrase: “locum”; any format (e.g., pdf, .xlsx, .doc)  Limited to: English language documents; India; without the words: nurse, job, ad, ads, advertisement, midwife, pharmacist  2) All these word(s): “locum”; any format (e.g., pdf, .xlsx, .doc)  Limited to: English language documents; India; without the words: nurse, job, ad, ads, advertisement, midwife, pharmacist  3) All these word(s): “locum”; any format (e.g., pdf, .xlsx, .doc)  4) This exact word or phrase: “contract doctor”; any format (e.g., pdf, .xlsx, .doc)  Limited to: English language documents; India  5) This exact word or phrase: “contract doctor”; any format (e.g., pdf, .xlsx, .doc)  Selection:  All items retrieved with each search were reviewed for relevance by 1 reviewer.  Items were selected by scanning the first 150 results from each search. | 1) 0  2) 0  3) 0  4) 1  5) 49 | 1) 0  2) 0  3) 0  4) 0  5) 0 |
| 21Jun2023 | IPH Bengaluru;  iphindia.org/ | Used Advanced Google site/domain search  Search strategies:  Search region limited to: India  1) This exact word or phrase: “locum”; any format (e.g., pdf, .xlsx, .doc)  Limited to: English language documents; India; without the words: nurse, job, ad, ads, advertisement, midwife, pharmacist  2) All these word(s): “locum”; any format (e.g., pdf, .xlsx, .doc)  Limited to: English language documents; India; without the words: nurse, job, ad, ads, advertisement, midwife, pharmacist  3) All these word(s): “locum”; any format (e.g., pdf, .xlsx, .doc)  4) This exact word or phrase: “contract doctor”; any format (e.g., pdf, .xlsx, .doc)  Limited to: English language documents; India  5) This exact word or phrase: “contract doctor”; any format (e.g., pdf, .xlsx, .doc)  Selection:  All items retrieved with each search were reviewed for relevance by 1 reviewer.  Items were selected by scanning the first 150 results from each search. | 1) 0  2) 0  3) 0  4) 32  5) 29 | 1) 0  2) 0  3) 0  4) 0  5) 0 |
| 22Jun2023 | Health Direct; healthdirect.gov.au/ | Used Advanced Google site/domain search  Search strategies:  Search region limited to: Australia  1) This exact word or phrase: “locum”; any format (e.g., pdf, .xlsx, .doc)  Limited to: English language documents; Australia; without the words: nurse, job, ad, ads, advertisement, midwife, pharmacist  2) All these word(s): “locum”; any format (e.g., pdf, .xlsx, .doc)  Limited to: English language documents; Australia; without the words: nurse, job, ad, ads, advertisement, midwife, pharmacist  3) All these word(s): “locum”; any format (e.g., pdf, .xlsx, .doc)  Selection:  All items retrieved with each search were reviewed for relevance by 1 reviewer.  Items were selected by scanning the first 150 results from each search. | 1) 142  2) 261  3) 365 | 1) 0  2) 0  3) 0 |
| 22Jun2023 | Australian Institute of Health and Welfare; aihw.gov.au | Used Advanced Google site/domain search  Search strategies:  Search region limited to: Australia  1) This exact word or phrase: “locum”; any format (e.g., pdf, .xlsx, .doc)  Limited to: English language documents; Australia; without the words: nurse, job, ad, ads, advertisement, midwife, pharmacist  2) All these word(s): “locum”; any format (e.g., pdf, .xlsx, .doc)  Limited to: English language documents; Australia; without the words: nurse, job, ad, ads, advertisement, midwife, pharmacist  3) All these word(s): “locum”; any format (e.g., pdf, .xlsx, .doc)  Selection:  All items retrieved with each search were reviewed for relevance by 1 reviewer.  Items were selected by scanning the first 150 results from each search. | 1) 109  2) 92  3) 599 | 1) 1  2) 0  3) 0 |
| 22Jun2023 | Public Health Association Australia; phaa.net.au | Used Advanced Google site/domain search  Search strategies:  Search region limited to: Australia  1) This exact word or phrase: “locum”; any format (e.g., pdf, .xlsx, .doc)  Limited to: English language documents; Australia; without the words: nurse, job, ad, ads, advertisement, midwife, pharmacist  2) All these word(s): “locum”; any format (e.g., pdf, .xlsx, .doc)  Limited to: English language documents; Australia; without the words: nurse, job, ad, ads, advertisement, midwife, pharmacist  3) All these word(s): “locum”; any format (e.g., pdf, .xlsx, .doc)  4) This exact word or phrase: “contract doctor”; any format (e.g., pdf, .xlsx, .doc)  Limited to: English language documents; Australia  Selection:  All items retrieved with each search were reviewed for relevance by 1 reviewer.  Items were selected by scanning the first 150 results from each search. | 1) 0  2) 1  3) 4  4) 41 | 1) 0  2) 0  3) 0  4) 0 |
| 22Jun2023 | JPS Medical Recruitment; [jpsmedical.com.au](https://www.jpsmedical.com.au/) | Used Advanced Google site/domain search  Search strategies:  Search region limited to: Australia  1) This exact word or phrase: “locum”; any format (e.g., pdf, .xlsx, .doc)  Limited to: English language documents; Australia; without the words: nurse, job, ad, ads, advertisement, midwife, pharmacist  2) All these word(s): “locum”; any format (e.g., pdf, .xlsx, .doc)  Limited to: English language documents; Australia; without the words: nurse, job, ad, ads, advertisement, midwife, pharmacist  3) All these word(s): “locum”; any format (e.g., pdf, .xlsx, .doc)  Selection:  All items retrieved with each search were reviewed for relevance by 1 reviewer.  Items were selected by scanning the first 150 results from each search. | 1) 0  2) 0  3) 86 | 1) 0  2) 0  3) 1 |
| 22Jun2023 | National Rural Health Alliance; ruralhealth.org.au | Used Advanced Google site/domain search  Search strategies:  Search region limited to: Australia  1) This exact word or phrase: “locum”; any format (e.g., pdf, .xlsx, .doc)  Limited to: English language documents; Australia; without the words: nurse, job, ad, ads, advertisement, midwife, pharmacist  2) All these word(s): “locum”; any format (e.g., pdf, .xlsx, .doc)  Limited to: English language documents; Australia; without the words: nurse, job, ad, ads, advertisement, midwife, pharmacist  3) All these word(s): “locum”; any format (e.g., pdf, .xlsx, .doc)  Selection:  All items retrieved with each search were reviewed for relevance by 1 reviewer.  Items were selected by scanning the first 150 results from each search. | 1) 94  2) 141  3) 1180 | 1) 1  2) 1  3) 0 |
| 29Jun2023 | Australian College of Rural and Remote Medicine; [acrrm.org.au](https://www.acrrm.org.au/) | Used Advanced Google site/domain search  Search strategies:  Search region limited to: Australia  1) This exact word or phrase: “locum”; any format (e.g., pdf, .xlsx, .doc)  Limited to: English language documents; Australia; without the words: nurse, job, ad, ads, advertisement, midwife, pharmacist  2) All these word(s): “locum”; any format (e.g., pdf, .xlsx, .doc)  Limited to: English language documents; Australia; without the words: nurse, job, ad, ads, advertisement, midwife, pharmacist  3) All these word(s): “locum”; any format (e.g., pdf, .xlsx, .doc)  Selection:  All items retrieved with each search were reviewed for relevance by 1 reviewer.  Items were selected by scanning the first 150 results from each search. | 1) 59  2) 68  3) 155 | 1) 2  2) 0  3) 0 |

**Grey Literature Database Search (Strategy 2)**

| Date | Database name & website URL | Search strategy(s)/ words searched including (if applicable) how items were selected. | # items retrieved/ search results | # of items screened (uploaded to citation management software) |
| --- | --- | --- | --- | --- |
| 14Jun2023 | OpenGrey; <https://opengrey.eu/> | Search strategie(s):  1) Any field: “Locum”  2) Any field: “Contract”  3) Any field: “Temporary”  4) Any field: “Physician”  5) Any field: “Rural physician”  Selection:  Items were selected by scanning the first 250 results from each search. | 1) 0  2) 186  3) 204  4) 106  5) 2 | 1) 0  2) 0  3) 0  4) 0  5) 0 |
| 14Jun2023 | Health Quality Ontario; <https://www.hqontario.ca/Evidence-to-Improve-Care/Health-Technology-Assessment> | Search strategie(s):  1) Any field: “Locum”  2) Any field: “Locum AND physician”  3) Any field: “Physician”  4) Any field: “Temporary”  5) Any field: “Temporary and Physician”  6) Any field: “Rural and Physician”  7) Any field: “Rural and Locum”  Selection:  Items were selected by scanning the first 250 results from each search. | 1) 5  2) 4  3) 3489  4) 121  5) 104  6) 458  7) 2 | 1) 1  2) 0  3) 0  4) 0  5) 0  6) 0  7) 0 |
| 14Jun2023 | Canadian Institute for Health Information; <https://www.cihi.ca/en> | Search strategie(s):  1) Any field: “Locum”  2) Any field: “Locum AND physician”  3) Any field: “Physician”  4) Any field: “Temporary”  5) Any field: “Temporary and Physician”  6) Any field: “Rural and Physician”  7) Any field: “Rural and Locum”  Selection:  Items were selected by scanning the first 250 results from each search. | 1) 21  2) 21  3) 573  4) 115  5) 74  6) 131  7) 12 | 1) 3  2) 0  3) 0  4) 0  5) 0  6) 0  7) 0 |
| 15Jun2023 | Scopus; <https://www-scopus-com.proxy.bib.uottawa.ca/search/form.uri?display=basic&zone=header&origin=#basic> | Search strategie(s):  1) Any field: “Locum”  Limited to: English language documents; 1990 or above; Medicine; UK, USA, Canada, India, Australia; …  Full search parameters:  ALL ( locum ) AND PUBYEAR > 1989 AND ( LIMIT-TO ( DOCTYPE , "le" ) OR LIMIT-TO ( DOCTYPE , "ch" ) OR LIMIT-TO ( DOCTYPE , "no" ) OR LIMIT-TO ( DOCTYPE , "cp" ) OR LIMIT-TO ( DOCTYPE , "sh" ) OR LIMIT-TO ( DOCTYPE , "ed" ) OR LIMIT-TO ( DOCTYPE , "er" ) ) AND ( LIMIT-TO ( LANGUAGE , "English" ) ) AND ( LIMIT-TO ( AFFILCOUNTRY , "United Kingdom" ) OR LIMIT-TO ( AFFILCOUNTRY , "United States" ) OR LIMIT-TO ( AFFILCOUNTRY , "Australia" ) OR LIMIT-TO ( AFFILCOUNTRY , "Canada" ) OR LIMIT-TO ( AFFILCOUNTRY , "India" ) ) AND ( LIMIT-TO ( SUBJAREA , "MEDI" ) OR LIMIT-TO ( SUBJAREA , "SOCI" ) OR LIMIT-TO ( SUBJAREA , "ARTS" ) OR LIMIT-TO ( SUBJAREA , "BUSI" ) OR LIMIT-TO ( SUBJAREA , "ECON" ) )  Selection:  Items were selected by scanning the first 250 results from each search. | 1) 229 | 1) 8 |
| 15Jun2023 | Agency for Healthcare Research and Quality; <https://datatools.ahrq.gov/hcupnet> | Search strategie(s):  1) Any field: “Locum”  Selection:  Items were selected by scanning the first 250 results from each search. | 1) 66 | 1) 0 |
| 16Jun2023 | Google Scholar; <https://scholar-google-com.proxy.bib.uottawa.ca/> | Search strategie(s):  1) Any field: “Locum”  Limited to: 1990 or above; without the words: nurse, job, ad, ads, advertisement, midwife  Selection:  Items were selected by scanning the first 150 results from each search. | 1) 16900 | 1) 19 |
| 17Jun2023 | MedNar; <https://mednar.com/mednar/desktop/en/search.html> | Search strategie(s):  1) Any field: “Locum”  Limited to: 1990 or above  Selection:  Items were selected by scanning the first 250 results from each search. | 1) 119 | 1) 6 |

**Search Engine Searching (Strategy 3)**

| Date | Search engine | Search strategy(s)/ words searched including (if applicable) how items were selected. | # items retrieved/ search results | # of items screened (uploaded to citation management software) |
| --- | --- | --- | --- | --- |
| 23Jun2023  15Jul2023  16Jul2023 | google.ca | Used Advanced Google site/domain search  Search strategie(s):  1) This exact word or phrase: “locum”; any format (e.g., pdf, .xlsx, .doc)  Limited to: English language documents; Canada; without the words: nurse, job, ad, ads, advertisement, midwife, pharmacist  2) This exact word or phrase: “locum”; pdf format  Limited to: English language documents; Canada; without the words: nurse, job, ad, ads, advertisement, midwife, pharmacist  3) This exact word or phrase: “locum physician”; any format (e.g., pdf, .xlsx, .doc)  Limited to: English language documents; Canada; without the words: nurse, job, ad, ads, advertisement, midwife, pharmacist  4) All these word(s): “locum”; any format (e.g., pdf, .xlsx, .doc)  Limited to: English language documents; Canada; without the words: nurse, job, ad, ads, advertisement, midwife, pharmacist  5) All these word(s): “locum”; pdf format  Limited to: English language documents; Canada; without the words: nurse, job, ad, ads, advertisement, midwife, pharmacist  6) All these word(s): “locum”; pdf format  7) All these word(s): “locum” “recruitment” “needs” “assessment”; pdf format  8) All these word(s): “locum” “physician” “recruitment”; pdf format  9) All these word(s): “locum” “recruitment”; any format (e.g., pdf, .xlsx, .doc)  10) All these word(s): “locum” “recruitment” “assessment”; any format (e.g., pdf, .xlsx, .doc)  Selection:  Items were selected by scanning the first 250 results from each search. | 1) 26400  2) 3390  3) 2090  4) 35500  5) 3910  6) 1270000  7) 82600  8) 43300  9) 5330000  10) 2040000 | 1) 5  2) 3  3) 1  4) 0  5) 0  6) 1  7) 5  8) 3  9) 1  10) 0 |
| 26Jun2023 | google.uk | Used Advanced Google site/domain search  Search strategie(s):  1) This exact word or phrase: “locum”; any format (e.g., pdf, .xlsx, .doc)  Limited to: English language documents; United Kingdom; without the words: nurse, job, ad, ads, advertisement, midwife, pharmacist  2) This exact word or phrase: “locum”; pdf format  Limited to: English language documents; United Kingdom; without the words: nurse, job, ad, ads, advertisement, midwife, pharmacist  3) This exact word or phrase: “sessional GP”; any format (e.g., pdf, .xlsx, .doc)  Limited to: English language documents; United Kingdom; without the words: nurse, job, ad, ads, advertisement, midwife, pharmacist  4) All these word(s): “locum”; any format (e.g., pdf, .xlsx, .doc)  Limited to: English language documents; United Kingdom; without the words: nurse, job, ad, ads, advertisement, midwife, pharmacist  5) All these word(s): “locum”; pdf format  Limited to: English language documents; United Kingdom; without the words: nurse, job, ad, ads, advertisement, midwife, pharmacist  Selection:  Items were selected by scanning the first 150 results from each search. | 1) 270000  2) 24800  3) 4210  4) 309000  5) 29600 | 1) 7  2) 8  3) 3  4) 0  5) 0 |
| 27Jun2023 | google.com | Used Advanced Google site/domain search  Search strategie(s):  1) This exact word or phrase: “locum”; any format (e.g., pdf, .xlsx, .doc)  Limited to: English language documents; USA; without the words: nurse, job, ad, ads, advertisement, midwife, pharmacist  2) This exact word or phrase: “locum”; pdf format  Limited to: English language documents; USA; without the words: nurse, job, ad, ads, advertisement, midwife, pharmacist  3) This exact word or phrase: “locum recruitment”; any format (e.g., pdf, .xlsx, .doc)  Limited to: English language documents; USA; without the words: nurse, job, ad, ads, advertisement, midwife, pharmacist  4) This exact word or phrase: “locum retention”; any format (e.g., pdf, .xlsx, .doc)  Limited to: English language documents; USA; without the words: nurse, job, ad, ads, advertisement, midwife, pharmacist  5) This exact word or phrase: “locum physician”; any format (e.g., pdf, .xlsx, .doc)  Limited to: English language documents; USA; without the words: nurse, job, ad, ads, advertisement, midwife, pharmacist  6) This exact word or phrase: “locum physician”; pdf format  Limited to: English language documents; USA; without the words: nurse, job, ad, ads, advertisement, midwife, pharmacist  7) All these word(s): “locum”; any format (e.g., pdf, .xlsx, .doc)  Limited to: English language documents; USA  8) All these word(s): “locum”; pdf format  Limited to: English language documents; USA  Selection:  Items were selected by scanning the first 150 results from each search. | 1) 190000  2) 8160  3) 5250  4) 53300  5) 11600  6) 135  7) 4100000  8) 96000 | 1) 0  2) 0  3) 0  4) 0  5) 0  6) 2  7) 0  8) 2 |
| 28Jun2023 | google.in | Used Advanced Google site/domain search  Search strategie(s):  1) This exact word or phrase: “locum”; any format (e.g., pdf, .xlsx, .doc)  Limited to: English language documents; India; without the words: nurse, job, ad, ads, advertisement, midwife, pharmacist  2) This exact word or phrase: “locum”; pdf format  Limited to: English language documents; India; without the words: nurse, job, ad, ads, advertisement, midwife, pharmacist  3) This exact word or phrase: “contract doctor”; any format (e.g., pdf, .xlsx, .doc)  Limited to: English language documents; India; without the words: nurse, job, ad, ads, advertisement, midwife, pharmacist  4) This exact word or phrase: “contract doctor”; pdf format  Limited to: English language documents; India; without the words: nurse, job, ad, ads, advertisement, midwife, pharmacist  5) All these word(s): “locum”; any format (e.g., pdf, .xlsx, .doc)  Limited to: English language documents; India; without the words: nurse, job, ad, ads, advertisement, midwife, pharmacist  6) All these word(s): “locum”; pdf format  Limited to: English language documents; India; without the words: nurse, job, ad, ads, advertisement, midwife, pharmacist  Selection:  Items were selected by scanning the first 150 results from each search. | 1) 56000  2) 873  3) 1080  4) 106  5) 80800  6) 829 | 1) 0  2) 0  3) 0  4) 0  5) 0  6) 0 |
| 29Jun2023 | google.au | Used Advanced Google site/domain search  Search strategie(s):  1) This exact word or phrase: “locum”; any format (e.g., pdf, .xlsx, .doc)  Limited to: English language documents; Australia; without the words: nurse, job, ad, ads, advertisement, midwife, pharmacist  2) This exact word or phrase: “locum”; pdf format  Limited to: English language documents; Australia; without the words: nurse, job, ad, ads, advertisement, midwife, pharmacist  3) All these word(s): “locum”; any format (e.g., pdf, .xlsx, .doc)  Limited to: English language documents; Australia; without the words: nurse, job, ad, ads, advertisement, midwife, pharmacist  4) All these word(s): “locum”; pdf format  Limited to: English language documents; Australia; without the words: nurse, job, ad, ads, advertisement, midwife, pharmacist  5) All these word(s): “locum”; any format (e.g., pdf, .xlsx, .doc)  Limited to: English language documents; Australia  6) All these word(s): “locum”; pdf format  Limited to: English language documents; Australia  Selection:  Items were selected by scanning the first 150 results from each search. | 1) 49300  2) 5950  3) 194000  4) 9050  5) 967000  6) 35400 | 1) 0  2) 0  3) 0  4) 0  5) 5  6) 2 |

**Conference Proceedings, Thesis/dissertations**

| Date | Database name & website URL | Search strategy(s)/ words searched including (if applicable) how items were selected. | # items retrieved/ search results | # of items screened (uploaded to citation management software) |
| --- | --- | --- | --- | --- |
| 15Jun2023 | Conference Alerts; <https://conferencealerts.com/> | Search strategie(s):  1) Any field: “Locum”  2) Any field: “Contract”  3) Any field: “Temporary”  4) Any field: “Temporary AND physician”  Selection:  Items were selected by scanning the first 250 results from each search. | 1) 0  2) 1  3) 17  4) 0 | 1) 0  2) 0  3) 0  4) 0 |
| 15Jun2023 | EBSCO (Open Dissertations); <https://www.ebsco.com/products/research-databases/ebsco-open-dissertations> | Search strategie(s):  1) Any field: “Locum”  Selection:  Items were selected by scanning the first 250 results from each search. | 1) 8 | 1) 0 |
| 15Jun2023 | ERIC; <https://eric.ed.gov/> | Search strategie(s):  1) Any field: “Locum”  Selection:  Items were selected by scanning the first 250 results from each search. | 1) 6 | 1) 1 |
| 15Jun2023 | Proquest (Dissertations and Theses Global); <https://www.proquest.com/pqdtglobal?_ga=2.77278485.1605536652.1686844991-1434983896.1686844991> | Search strategie(s):  1) Any field: “Locum”  Limited to: English language documents; 1990 or above  2) Any field: “Locum physician”  Limited to: English language documents; 1990 or above  Selection:  Items were selected by scanning the first 250 results from each search. | 1) 5815  2) 18 | 1) 3  2) 0 |
| 15Jun2023 | WorldCatDissertations; <https://www.worldcat.org/> | Search strategie(s):  1) Any field: “Locum”  Limited to: English language documents; 1990 or above; theses or dissertations  Selection:  Items were selected by scanning the first 250 results from each search. | 1) 27 | 1) 0 |
| 15Jun2023 | Scopus; <https://www-scopus-com.proxy.bib.uottawa.ca/search/form.uri?display=basic&zone=header&origin=#basic> | Search strategie(s):  1) Any field: “Locum”  Limited to: English language documents; 1990 or above; conference proceedings  Selection:  Items were selected by scanning the first 250 results from each search. | 1) 7 | 1) 1 |
| 16Jun2023 | Theses Canada; <https://library-archives.canada.ca/eng/services/services-libraries/theses/Pages/search-theses-canada.aspx> | Search strategie(s):  1) Any field: “Locum”  Limited to: English language documents  2) Any field: “Locum physician”  Limited to: English language documents  3) Any field: “Contract physician”  Limited to: English language documents  Selection:  Items were selected by scanning the first 250 results from each search. | 1) 0  2) 0  3) 588 | 1) 0  2) 0  3) 0 |

**Contact of Knowledge Experts**

| Name | Date contacted | # of items recommended | # of items identified for full screening (uploaded to citation management software) |
| --- | --- | --- | --- |
| Dr. Eli Orrantia | 5-Jul-2023 | 2 | 2 |
